# Genome‐wide analysis of HD‐Zip genes in *Sophora alopecuroides* and their role in salt stress response

**DOI:** 10.1002/tpg2.20504

**Published:** 2024-08-28

**Authors:** Youcheng Zhu, Di Wang, Fan Yan, Le Wang, Ying Wang, Jingwen Li, Xuguang Yang, Ziwei Gao, Xu Liu, Yajing Liu, Qingyu Wang

**Affiliations:** ^1^ College of Biological and Agricultural Engineering Jilin University Changchun China; ^2^ College of Plant Science Jilin University Changchun China

## Abstract

We aimed to identify HD‐Zip (homologous domain leucine zipper) family genes based on the complete *Sophora alopecuroides* genome sequence. Eighty‐six *Sophora alopecuroides* HD‐Zip family (*SaHDZ*) genes were identified and categorized into four subclasses using phylogenetic analysis. Chromosome localization analysis revealed that these genes were distributed across 18 chromosomes. Gene structure and conserved motif analysis showed high similarity among members of the *SaHDZ* genes. Prediction analysis revealed 71 cis‐acting elements in *SaHDZ* genes. Transcriptome and quantitative real‐time polymerase chain reaction analyses showed that under salt stress, *SaHDZ* responded positively in *S. alopecuroides*, and that SaHDZ22 was significantly upregulated afterward. Functional verification experiments revealed that *SaHDZ22* overexpression increased the tolerance of *Arabidopsis* to salt and osmotic stress. Combined with cis‐acting element prediction and expression level analysis, HD‐Zip family transcription factors may be involved in regulating the balance between plant growth and stress resistance under salt stress by modulating the expression of auxin and abscisic acid signaling pathway genes. The *Sophora alopecuroides* adenylate kinase protein (*SaAKI*) and *S. alopecuroides* tetrapeptide‐like repeat protein (SaTPR; pCAMBIA1300‐SaTPR‐cLUC) expression levels were consistent with those of *SaHDZ22*, indicating that SaHDZ22 may coordinate with SaAKI and SaTPR to regulate plant salt tolerance. These results lay a foundation in understanding the salt stress response mechanisms of *S*. *alopecuroides* and provide a reference for future studies oriented toward exploring plant stress resistance.

## Core Ideas

- A total of 86 *SaHDZ* (*Sophora alopecuroides* HD‐Zip family) genes were identified and characterized.
- Phylogenetic topology and conserved domains were used to define four subgroups.
- *SaHDZ* genes play crucial roles in salt stress response in *S. alopecuroides*.
- Overexpressed *SaHDZ22* increased *Arabidopsis* tolerance to salt stress and osmotic stress.
- SaHDZ22 may coordinate with *S*. *alopecuroides* adenylate kinase protein and SaTPR to regulate plant salt tolerance.

**Abbreviations**
ABA: abscisic acid
AD: pGADT7‐cDNA
AUX: auxin
BD: pGBKT7‐*SaHDZ22*
GL2: GLABRA2
HALZ: homeobox associated leucine zipper
HB: homeobox
HD: homeodomain
HD‐Zip: homologous domain leucine zipper
JA‐Ile: jasmonate‐isoleucine
LZ: leucine
MEKHLA: methionine‐glutamic‐lysine‐histidine‐leucine‐alanine
ROS: reactive oxygen species
SaAKI: *Sophora alopecuroides* adenylate kinase protein
SAG113: probable protein phosphatase 2C 78
*SaHDZ*
: *Sophora alopecuroides* HD‐Zip family
START: steroidogenic acute regulatory protein‐related lipid transfer
TPR: tetra tripeptide repeat

## 1 INTRODUCTION

To date, homologous domain leucine zipper (HD‐Zip) transcription factors have only been found in plants; they are considered plant‐specific transcription factors characterized by the existence of a homeodomain (HD) and leucine (LZ), which are closely related to the LZ zipper sequence (Henriksson et al., 2005). HD‐Zip family genes are divided into four subfamilies (I–IV) based on the sequence homology of their HD‐Zip domain, conserved sequences, and specific intron/exon locations (Ariel et al., 2007; Ciarbelli et al., 2008; Henriksson et al., 2005; Nakamura et al., 2006). HD‐Zip I and II genes share structural similarities, including a highly conserved HD domain and relatively conserved LZ domain; however, some distinct elements exist between class I and II genes, enabling them to be differentiated (Y. Li, Yang, et al., 2022). HD‐Zip III and IV contain an additional steroidogenic acute regulatory protein‐related lipid transfer (START) domain and conserved START‐associated domain (Zhu et al., 2018; Y. Li, Yang, et al., 2022). HD‐Zip III family genes also contain a highly conserved methionine‐glutamic‐lysine‐histidine‐leucine‐alanine (MEKHLA) domain (Zhu et al., 2018). HD‐Zip transcription factor family genes are considered key for crop improvement due to their roles in plant growth, environmental stress, and environmental adaptation, and are of considerable research interest.

The HD‐Zip transcription factor family is one of the largest plant‐specific superfamilies and plays important roles in abiotic stress responses. ATHB‐7, an HD‐Zip I transcription factor family member in *Arabidopsis thaliana*, has been demonstrated to be strongly induced by drought and exogenous abscisic acid (ABA) and is involved in the transcriptional regulation of the drought response signaling pathway in an ABA‐dependent manner (Söderman et al., 1996). To date, HD‐Zip transcription factors have been extensively explored and functionally verified in rice (S. Zhang et al., 2012), wheat (Kovalchuk et al., 2016), tobacco (Ré et al., 2011), alfalfa (Ariel et al., 2010), corn (Y. Zhao et al., 2014), soybean (Belamkar et al., 2014), chili (Mou et al., 2017), apple (Jiang et al., 2017), cotton (He et al., 2018), litchi (lychee) (C. Li, Zhao, et al., 2019), birch (Tan et al., 2020), sweet cherry (Qiu et al., 2021), and *Lotus japonicus* (D. Wang, Gong, et al., 2022) plants. In wheat, the *TaHDZipI‐2* gene plays a role in several key processes that control frost tolerance, flowering transition, and panicle development, and its overexpression can improve plant frost tolerance (Kovalchuk et al., 2016). AtHB12 induces root elongation and leaf development in *Arabidopsis* seedlings under normal growth conditions, whereas AtHB7 promotes leaf development, increases chlorophyll levels, enhances photosynthesis, and decreases stomatal conductivity in mature plants (Ré et al., 2014). Overexpression of the rice HD‐Zip I transcription factor gene *OsTF1L* significantly improves drought tolerance during the vegetative stage of growth and also promotes effective photosynthesis and water retention under drought conditions, which are key regulators of drought tolerance (Bang et al., 2019).

The HD‐Zip transcription factor family plays important roles in plant responses to abiotic stress.

Under salt stress and ABA treatment, the alfalfa HD‐Zip I transcription factor MtHB1 protein weakened AUX signaling by inhibiting LBD1 (a transcription factor) and decreasing lateral root development (Ariel et al., 2010). *Oshox22* expression in rice is strongly induced by treatment with salt, ABA, and polyethylene glycol. Oshox22 affects ABA biosynthesis and regulates drought and salt stress responses via ABA‐mediated signal transduction pathways (S. Zhang et al., 2012). *LjHDZ7* overexpression in *L*. *japonicus* increased the resistance of transgenic *Arabidopsis* plants to salt stress (D. Wang, Gong, et al., 2022). *BpHOX2* (a HD‐Zip I gene) expression in *Betula platyphylla* was induced by osmosis and salt, and its overexpression significantly improved the osmotic tolerance of *B. platyphylla* plants, whereas *BpHOX2* knockout increased their sensitivity to osmotic stress (Tan et al., 2020). *BpHOX2* expression improved proline levels and reactive oxygen scavenging capacity by inducing the expression of pyrroline‐5‐carboxylate synthase, peroxidase, and superoxide dismutase‐related genes (Tan et al., 2020). *MdHB7* (a HD‐Zip I gene) expression in *Malus domestica* was induced by salt stress, and MdHB7 overexpression improved the photosynthetic performance of *M. domestica* plants while preventing reactive oxygen species (ROS) and Na^+^ accumulation under salt stress (S. Zhao et al., 2021). MdHB7‐like protein binds to the *Mdccx1* promoter and promotes its expression, thereby directly promoting the efflux of Na^+^ under salt stress via an autophagy‐independent pathway and enhancing salt tolerance in apples (J. Yang et al., 2023). *Arabidopsis* HD‐Zip I transcription factors AtHB23, AtPHL1, and AtMYB68 interact with each other to regulate plant root development and responses to salt stress (Spies et al., 2023). Functional verification of HD‐Zip I transcription factor family genes has shown that they are mainly involved in plant responses to abiotic stress, but the pathway and specific mechanisms underlying their involvement require further investigations.

*Sophora alopecuroides* is a small perennial shrub found in northwest China that has important medicinal value and is highly adaptable to abiotic stresses (salt, alkali, and drought) (Yan et al., 2020). As an ideal gene resource for stress resistance, we screened several *S. alopecuroides* stress resistance‐related genes, including HD‐Zip transcription factor family proteins. As there are no studies on the HD‐Zip family genes of *S. alopecuroides*, we used the complete whole genome and transcriptome data of *S. alopecuroides* to identify the HD‐Zip transcription factor family genes, explore the contribution of HD‐Zip family proteins to resistance, and construct an interaction network of HD‐Zip proteins. The function of the HD‐Zip I subfamily gene *SaHDZ22*, where SaHDZ is *Sophora alopecuroides* HD‐Zip family, in *A*. *thaliana* was verified and the potential mechanism of action was explored. Our findings will provide a reference for understanding the mechanisms of stress adaptability in *S. alopecuroides* and a new genetic resource for further genetic improvement of crop stress adaptability.

**Core Ideas**

- A total of 86 *SaHDZ* (*Sophora alopecuroides* HD‐Zip family) genes were identified and characterized.
- Phylogenetic topology and conserved domains were used to define four subgroups.
- *SaHDZ* genes play crucial roles in salt stress response in *S. alopecuroides*.
- Overexpressed *SaHDZ22* increased *Arabidopsis* tolerance to salt stress and osmotic stress.
- SaHDZ22 may coordinate with *S*. *alopecuroides* adenylate kinase protein and SaTPR to regulate plant salt tolerance.

## 2 MATERIALS AND METHODS

### 2.1 Plant materials

The *S. alopecuroides* seeds were collected from the Korla region of Xinjiang (China). Seeding, germination, and sampling of *S*. *alopecuroides*, including treatment of the seed coats with 98% concentrated sulfuric acid prior to seeding to promote germination, were performed in accordance with previous studies (Yan et al., 2020). Seeding was performed in turf soil consisting of vermiculite (3:1), and the plants were cultured in an AI climate chamber (day/night cycle: 16 h/8 h, temperature: 26°C/22°C, humidity: 65%, light intensity: 30000 Lux). Four‐week‐old *S. alopecuroides* seedlings were treated with a Hoagland nutrient solution containing 1.2% NaCl to simulate salt stress. Plant samples were collected at 0, 4, 24, 48, and 72 h after salt stress and frozen in liquid nitrogen at −80°C.

### 2.2 Genome‐wide identification of the HD‐Zip gene family of *S. alopecuroides*

Along with the previously annotated whole‐genome information database of *S*. *alopecuroides*, we used a combination of BLAST (Basic Local Alignment Search Tool) and HMM (Hidden Markov Model) to identify HD‐Zip family genes. First, BLAST was used to compare *S. alopecuroides* sequences with the HD‐Zip protein sequences of *Arabidopsis* and soybeans, and HMM was used to predict the protein domains based on conserved HD (PF00046) and LZ (PF02183) domains. HD‐Zip candidate protein sequences were identified by CD Search.

### 2.3 Multiple sequence alignment and phylogenetic tree analysis of HD‐Zip genes in *S. alopecuroides*

The HD‐Zip protein sequences of *A. thaliana* and soybeans were obtained from previous studies (Belamkar et al., 2014; Chen et al., 2014). The MUSCLE MEGAX program was used to analyze the HD‐Zip amino acid sequences from *A. thaliana*, soybeans, and *S. alopecuroides*. A phylogenetic tree was constructed using the maximum likelihood method (with 1000 bootstrap replicates). The phylogenetic trees were modified using iTOL online software (https://itol.embl.de/). HD‐Zip sequence information is shown in Table S1.

### 2.4 Chromosome localization analysis

Gff files were used to obtain gene location information, which included chromosome numbers and base intervals. The location of the gene of interest on the full‐length chromosome was then determined.

### 2.5 Gene structure, conserved domain, and cis‐regulatory element analysis

The exon and intron locations of *SaHDZ* genes were extracted from the annotated genome file and visualized directly with TBtools. The SaHDZ genetic structure was drawn using TBtools for conservative structure domain analysis and visualization (https://www.ncbi.nlm.nih.gov/Structure/bwrpsb/bwrpsb.cg). The protein sequences of SaHDZ members were submitted to the MEME program for analysis of conserved motifs. The 2000 bp sequence (upstream of the start codon) of all *SaHDZ* genes was used for cis‐regulatory element analysis using the PlantCARE database (http://bioinformatics.psb.ugent.be/webtools/plantcare/html/). In addition, key auxin (AUX) and ABA signaling pathway promoters were obtained using the whole genome data of *S. alopecuroides* to conduct cis‐acting element analyses.

### 2.6 Conserved analysis and interaction network of HD‐Zip proteins

DNAMAN 6.0.3.99 was used to analyze the sequence conservation of HD‐Zip family proteins in *S. alopecuroides*. STRING (https://cn.string‐db.org/) was used to calculate potential interactions between SaHDZ proteins. The protein sequences of *SaHDZ* genes were uploaded to STRING, and the homologous genes in *Arabidopsis*/soybeans were selected to establish the protein interaction network.

### 2.7 SaHDZ gene expression pattern analysis

To explore the expression patterns of *SaHDZ* genes in different tissues, we extracted RNA from the roots, stems, and leaves of the 4‐week‐old *S. alopecuroides* for transcriptome sequencing. To investigate the response of *SaHDZ* genes to salt stress, transcriptome sequencing was performed on the root tissues of *S. alopecuroides* at different time points under salt stress. R software was used to determine the expression levels of SaHDZ transcripts, and the fragments per kilobase of exon per million fragments mapped values were used to develop the expression profile heatmap, which was visualized using TBtools. Eight *SaHDZ* genes were randomly selected, and their expression levels were further verified by quantitative real‐time polymerase chain reaction (qRT‐PCR) after salt stress. Reverse transcription was performed using StarScript III RT MasterMix (A232‐10; Beijing Kangrun Chengye Biotechnology Co., Ltd.), according to the manufacturer's instructions. qRT‐PCR was performed using SYBR Green Real‐time PCR Mix (Thermo Fisher Scientific) on a CFX Connect Real‐Time PCR System (Bio‐Rad). The primers used are listed in Table S2.

### 2.8 Cloning and vector construction of the *SaHDZ22* gene

The nucleic acid sequence of *SaHDZ22* was obtained by combining genome and transcriptome sequencing results. Using Primer 6.0 to design primers, *SaHDZ22* was amplified using a high‐fidelity enzyme with *S. alopecuroides* cDNA as the template. The coding sequence of *SaHDZ22* was recombined into the plant expression vector pCAMBIA3301‐GFP and pCHF3300, and yeast expression vector pGBKT7 using double enzyme digestion. The nucleic acid sequence of SaHDZ22 was divided into the N‐terminal, HB (homeobox), HALZ (Homeobox associated leucine zipper), and C‐terminal (Table S3) regions, combined, and then recombined into the yeast vector pGBKT7 using double enzyme digestion to analyze the self‐activation activity of SaHDZ22. The inserted fragments were verified using monoclonal colony PCR and sequencing.

### 2.9 Transformation of SaHDZ22 into *Arabidopsis* and analysis for salt tolerance

*Arabidopsis* was cultured to the four‐leaf stage and transplanted into potting soil (turf soil: vermiculite = 1:1), and cultured in the culture room (day/night, 16 h/8 h, 22°C/20°C) for 3 weeks until infection and transformation. *Arabidopsis* was infected by *Agrobacterium tumefaciens* mediated dip‐flower method. The seeds of T_0_ generation were harvested, screened, and identified until the seeds of T_2_ generation were obtained for the next experiment.

The seeds of T_2_ overexpressing lines and wild‐type *Arabidopsis* were disinfected and sown on MS (Murashige Skoog) solid medium with CK (Control), 100 mM NaCl, and 200 mM mannitol and vernalized for 72 h at 4°C. The germination rate was counted after 3 days of culture, the survival rate was counted after 7 days, and the root length was counted after 15 days. The treatment experiment was repeated three times in parallel each time. During the germination period of *Arabidopsis*, timely analysis of germination, photographs, root length measurement, and statistics were carried out. The seeds of wild type (WT) and OE (overexpressed strain of *SaHDZ22*) *Arabidopsis* were scattered in a hole tray filled with culture soil (turf soil; vermiculite = 1:1). After 3 weeks of culture, plants were watered with 1/2 × Hoagland nutrient solution containing 200 mM NaCl. Wild type and transgenic strains treated with the control and 200 mM NaCl were subjected to RNA extraction and was reverse‐transcribed using reverse transcription‐quantitative polymerase chain eaction to analyze key genes involved in the AUX and ABA signal transduction pathways (refer to Section 2.7).

### 2.10 Transcriptome analysis of AUX and ABA signal transduction pathways in response to salt stress in *S. alopecuroides*

Based on transcriptome and genome data, target genes were screened using knockout annotation results. AUX signaling pathway genes included AUX influx carrier (AUX1; K13946), transport inhibitor response 1 (TIR1; K14485), AUX/AUX‐responsive protein IAA (AUX/IAA; K14484), AUX response factor (ARF; K14486), AUX responsive GH3 gene family (GH3; K14487), and SAUR family protein (SAUR; K14488). ABA signal transduction pathway genes included ABA receptor PYR/PYL family (PYR/PYL; K14496), protein phosphatase 2C (PP2C; K14497), serine/threonine‐protein kinase SRK2 (SnRK2; K14498), and ABA responsive element binding factor (ABF; K14432). The transcript per kilobase per million mapped reads (TPM) value of the selected genes was calculated and used to analyze the expression trend. Genes with TPM value <1 and no significant difference in each stage of salt stress were manually removed. Results were visualized using Excel (Microsoft).

### 2.11 SaHDZ22 subcellular localization and self‐activating activity analysis

Agrobacterium EHA105 was used to transiently express the recombinant plasmid in infected tobacco to determine its subcellular localization. Four weeks after the tobacco seedlings were cultured, the bacterial solution (OD_600_ adjusted to 0.8–1.0) and suspension (OD_600_ of 0.5) were injected into the epidermis of tobacco leaves, using Hoechst staining for 10 min, and leaves were observed under a microscope and photographed. For the self‐activation experiment, the yeast Y2H receptor state was prepared, and the reaction was established. The yeast were subsequently transformed, and the coating plate was screened (SD/‐Trp/‐Leu /X‐α‐Gal/AbA) to observe whether the single colony had turned blue (indicating self‐activation activity). This technique was based on the experimental method described by Biotechnology.

### 2.12 Yeast two‐hybrid screening for interacting proteins

The mating method was used to screen the yeast nuclear system two‐hybrid library, and the monoclonal Y2Hgold containing the pGBKT7‐*SaHDZ22* (BD) plasmid was seeded in liquid medium and cultured to an OD_600_ of 0.8. pGADT7‐cDNA (AD) and BD were mixed and cultured for 24 h, spread on 40 plates, and then cultured at 30°C for 3–5 days. A yeast plasmid extraction kit was used to extract the plasmids, based on the methods mentioned in previous studies (Zhu et al., 2021). OeBiotech provided a one‐to‐one interaction verification method.

### 2.13 Luciferase complementation assays

Recombinant plasmids for SaHDZ22 (pCAMBIA1300‐*SaHDZ22*‐nLUC) and *S. alopecuroides* adenylate kinase protein (*Sophora alopecuroides* adenylate kinase protein [SaAKI]; pCAMBIA1300‐*SaAK*I‐cLUC), *S. alopecuroides* tetrapeptide‐like repeat protein (SaTPR; pCAMBIA1300‐*SaTPR*‐cLUC) were transformed into Agrobacterium EHA105 and used to infect the lower epidermis of tobacco leaves for instantaneous expression. The transfected leaves were cultured for 36–48 h prior to gently smearing 1 mM D‐fluorescein (103404‐75‐7; Sangon Biotech) evenly onto the leaves. After incubation in the dark for 5 min, imaging of the leaves was performed. The luciferase (LUC) signal was detected using a cooled charge‐coupled device camera (Nikon‐L936; Andor Tech) with an exposure time of 10–20 min. pCAMBIA1300‐nLUC and pCAMBIA1300‐cLUC were used as negative controls. qRT‐PCR (described in Section 2.7) was used to investigate the changes in expression of genes encoding the interacting proteins under salt stress.

### 2.14 Statistical analyses

All experiments included three independent biological replicates, and Duncan's multi‐interval test (*p* < 0.01) was performed on the obtained data using one‐way analysis of variance in SPSS 23 (IBM Corp.). The qRT‐PCR results were visualized using GraphPad Prism 9 and Microsoft Excel 2010.

## 3 RESULTS

### 3.1 Identification and classification of HD‐Zip genes in *S. alopecuroides*

We identified the HD‐Zip transcription factor family in *S. alopecuroides* using genomic data. A total of 86 HD‐Zip genes were identified and named according to their distribution on chromosomes (*SaHDZ1* to *SaHDZ86*). Protein size analysis showed that the *SaHDZ* genes contained 168–847 amino acids, with molecular weights ranging from 19.40 to 92.99 kDa, and isoelectric points (pls) ranging from 4.63 to 8.92 (Table 1). Subcellular localization prediction showed that all 86 SaHDZ family proteins in *S. alopecuroides* were localized in the nucleus (Table 1).

**TABLE 1:** Summary of 86 HD‐Zip (homologous domain leucine zipper) family genes in *S*. *alopecuroides*.

No.	Gene name	Gene ID	Subgroup	CDS length (bp)	Protein size (aa)	Molecular weight (KDa)	pI	Predicted localization
1	*SaHDZ1*	Sa01G011480	Clade II	870	289	32.08	8.84	Nucleus
2	*SaHDZ2*	Sa01G014270	Clade IV	2490	829	90.60	5.97	Nucleus
3	*SaHDZ3*	Sa01G033880	Clade IV	2343	780	85.44	6.06	Nucleus
4	*SaHDZ4*	Sa01G037750	Clade II	1119	372	42.00	6.48	Nucleus
5	*SaHDZ5*	Sa01G043100	Clade I	507	168	19.40	7.74	Nucleus
6	*SaHDZ6*	Sa01G048820	Clade IV	2304	767	83.37	5.62	Nucleus
7	*SaHDZ7*	Sa01G051800	Clade III	2532	843	92.10	5.99	Nucleus
8	*SaHDZ8*	Sa02G073750	Clade II	888	295	32.54	8.09	Nucleus
9	*SaHDZ9*	Sa02G076690	Clade IV	2538	845	92.15	5.94	Nucleus
10	*SaHDZ10*	Sa02G093630	Clade IV	2331	776	85.36	6.05	Nucleus
11	*SaHDZ11*	Sa02G096580	Clade II	1092	363	40.30	7.15	Nucleus
12	*SaHDZ12*	Sa02G102370	Clade I	534	177	20.46	6.97	Nucleus
13	*SaHDZ13*	Sa02G107460	Clade IV	2307	768	83.50	5.66	Nucleus
14	*SAHDZ14*	Sa02G110470	Clade III	2535	844	92.35	6.02	Nucleus
15	*SaHDZ15*	Sa03G146890	Clade I	855	284	32.25	5.78	Nucleus
16	*SaHDZ16*	Sa03G147500	Clade I	984	327	37.26	4.67	Nucleus
17	*SaHDZ17*	Sa03G148700	Clade IV	2502	833	90.69	5.83	Nucleus
18	*SaHDZ18*	Sa03G152110	Clade I	741	246	28.27	5.07	Nucleus
19	*SaHDZ19*	Sa04G197230	Clade I	840	279	31.59	6.02	Nucleus
20	*SaHDZ20*	Sa04G197800	Clade I	987	328	37.40	4.63	Nucleus
21	*SaHDZ21*	Sa04G199060	Clade IV	2511	836	91.31	5.92	Nucleus
22	*SaHDZ22*	Sa04G202270	Clade I	738	245	28.14	5.34	Nucleus
23	*SaHDZ23*	Sa05G226050	Clade II	873	290	32.59	7.07	Nucleus
24	*SaHDZ24*	Sa05G230470	Clade III	2523	840	92.33	6.00	Nucleus
25	*SaHDZ25*	Sa05G237340	Clade I	741	246	28.87	6.36	Nucleus
26	*SaHDZ26*	Sa05G264930	Clade I	954	317	35.98	5.15	Nucleus
27	*SaHDZ27*	Sa06G273000	Clade II	855	284	32.20	8.74	Nucleus
28	*SaHDZ28*	Sa06G277300	Clade III	2523	840	92.30	6.03	Nucleus
29	*SaHDZ29*	Sa06G284350	Clade I	744	247	29.06	6.08	Nucleus
30	*SaHDZ30*	Sa06G309310	Clade I	951	316	35.96	4.99	Nucleus
31	*SaHDZ31*	Sa07G311990	Clade IV	2166	721	79.64	7.57	Nucleus
32	*SaHDZ32*	Sa07G320460	Clade II	900	299	33.92	6.68	Nucleus
33	*SaHDZ33*	Sa07G322190	Clade II	876	291	32.47	8.58	Nucleus
34	*SaHDZ34*	Sa07G347370	Clade I	840	279	31.76	5.83	Nucleus
35	*SaHDZ35*	Sa07G355430	Clade IV	2427	808	89.88	5.43	Nucleus
36	*SaHDZ36*	Sa07G360450	Clade I	639	212	24.81	7.11	Nucleus
37	*SaHDZ37*	Sa07G362850	Clade II	924	307	33.99	7.54	Nucleus
38	*SaHDZ38*	Sa07G364110	Clade II	891	296	33.16	7.09	Nucleus
39	*SaHDZ39*	Sa07G368270	Clade IV	2205	734	80.64	5.99	Nucleus
40	*SaHDZ40*	Sa08G370090	Clade IV	2193	730	80.29	6.63	Nucleus
41	*SaHDZ41*	Sa08G378740	Clade II	900	299	33.67	7.66	Nucleus
42	*SaHDZ42*	Sa08G380320	Clade II	882	293	32.55	8.39	Nucleus
43	*SaHDZ43*	Sa08G400740	Clade I	849	282	32.09	5.84	Nucleus
44	*SaHDZ44*	Sa08G401050	Clade I	912	303	34.78	4.77	Nucleus
45	*SaHDZ45*	Sa08G408340	Clade IV	2430	809	90.10	5.51	Nucleus
46	*SaHDZ46*	Sa08G412960	Clade I	648	215	25.11	6.21	Nucleus
47	*SaHDZ47*	Sa08G415340	Clade II	846	281	31.09	6.99	Nucleus
48	*SaHDZ48*	Sa08G416540	Clade II	894	297	33.13	7.07	Nucleus
49	*SaHDZ49*	Sa08G420350	Clade IV	2226	741	81.44	6.04	Nucleus
50	*SaHDZ50*	Sa09G432670	Clade II	1014	337	36.87	8.84	Nucleus
51	*SaHDZ51*	Sa09G455190	Clade IV	1995	664	73.95	5.48	Nucleus
52	*SaHDZ52*	Sa09G455420	Clade III	2532	843	92.53	5.85	Nucleus
53	*SaHDZ53*	Sa10G479030	Clade II	987	328	35.50	8.69	Nucleus
54	*SaHDZ54*	Sa10G506180	Clade III	2520	839	92.08	5.61	Nucleus
55	*SaHDZ55*	Sa11G523130	Clade IV	2145	714	79.35	6.17	Nucleus
56	*SaHDZ56*	Sa11G529010	Clade II	798	265	29.57	8.05	Nucleus
57	*SaHDZ57*	Sa11G552010	Clade IV	2298	765	82.98	5.77	Nucleus
58	*SaHDZ58*	Sa11G554720	Clade III	2544	847	92.49	5.92	Nucleus
59	*SaHDZ59*	Sa11G558380	Clade IV	2301	766	85.19	6.13	Nucleus
60	*SaHDZ60*	Sa11G558800	Clade III	2526	841	92.82	6.24	Nucleus
61	*SaHDZ61*	Sa12G565150	Clade IV	2184	727	80.54	6.43	Nucleus
62	*SaHDZ62*	Sa12G571340	Clade II	786	261	29.66	8.31	Nucleus
63	*SaHDZ63*	Sa12G595590	Clade IV	2298	765	83.07	5.90	Nucleus
64	*SaHDZ64*	Sa12G598650	Clade III	2544	847	92.71	5.86	Nucleus
65	*SaHDZ65*	Sa12G602590	Clade III	2526	841	92.68	6.00	Nucleus
66	*SaHDZ66*	Sa12G602800	Clade IV	1914	637	70.61	6.36	Nucleus
67	*SaHDZ67*	Sa13G629800	Clade I	930	309	35.07	4.76	Nucleus
68	*SaHDZ68*	Sa13G635330	Clade I	975	324	36.21	4.64	Nucleus
69	*SaHDZ69*	Sa14G674730	Clade I	924	307	34.75	4.88	Nucleus
70	*SaHDZ70*	Sa14G680550	Clade I	972	323	36.19	4.72	Nucleus
71	*SaHDZ71*	Sa15G695940	Clade I	867	288	32.90	4.80	Nucleus
72	*SaHDZ72*	Sa15G697670	Clade I	861	286	32.83	6.73	Nucleus
73	*SaHDZ73*	Sa15G710450	Clade II	1059	352	38.75	8.63	Nucleus
74	*SaHDZ74*	Sa15G714720	Clade I	672	223	25.93	8.14	Nucleus
75	*SaHDZ75*	Sa15G737000	Clade IV	2433	810	88.22	5.83	Nucleus
76	*SaHDZ76*	Sa15G739230	Clade IV	2088	695	76.34	8.40	Nucleus
77	*SaHDZ77*	Sa16G747010	Clade I	867	288	32.93	4.74	Nucleus
78	*SaHDZ78*	Sa16G748980	Clade I	852	283	32.43	6.62	Nucleus
79	*SaHDZ79*	Sa16G763890	Clade II	1050	349	38.37	8.92	Nucleus
80	*SaHDz80*	Sa16G768280	Clade I	666	221	25.53	8.10	Nucleus
81	*SaHDZ81*	Sa16G792750	Clade IV	2478	825	90.32	5.90	Nucleus
82	*SaHDZ82*	Sa16G794550	Clade IV	2052	683	75.27	6.22	Nucleus
83	*SaHDZ83*	Sa17G805790	Clade III	2523	840	92.96	6.12	Nucleus
84	*SaHDZ84*	Sa17G816310	Clade II	1167	388	43.00	6.54	Nucleus
85	*SaHDZ85*	Sa18G840120	Clade III	2523	840	92.99	6.15	Nucleus
86	*SaHDZ86*	Sa18G850000	Clade II	1203	400	44.34	6.20	Nucleus

Abbreviations: CDS, coding sequence; pl, isoelectric point.

**FIGURE 1:**
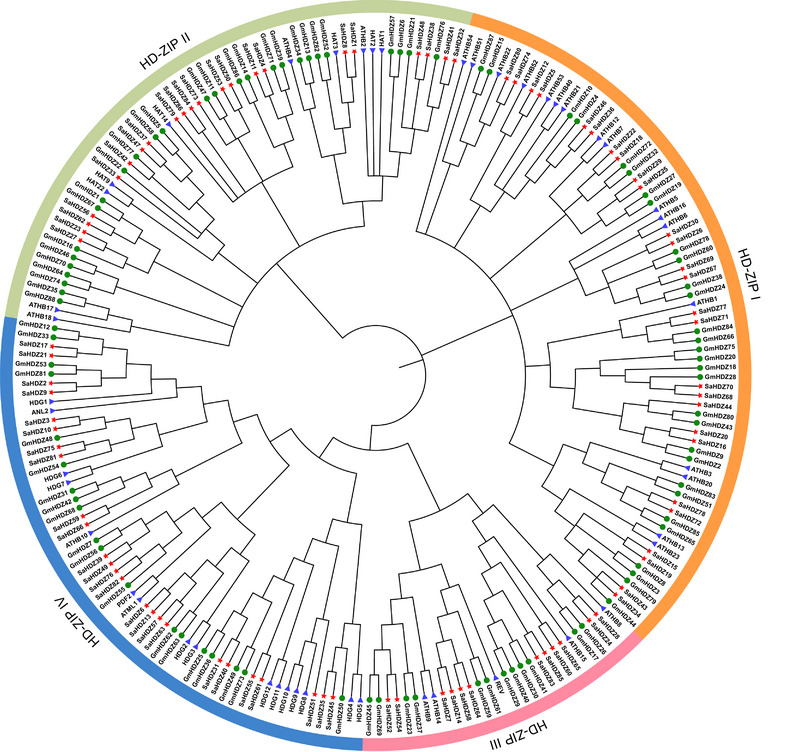
Phylogenetic analysis of HD‐Zip (homologous domain leucine zipper) proteins from *S*. *alopecuroides*, *A*. *thaliana*, and *Glycine max* (L.). The red star represents *S*. *alopecuroides* genes, blue triangle represents *Arabidopsis* genes, and green circle represents soybean genes.

We used the amino acid sequences of 222 HD‐Zip proteins from *Arabidopsis*, soybeans, and *S. alopecuroides* to generate phylogenetic trees. The results showed that the 86 *SaHDZ* genes could be divided into four subclasses (I: 27, II: 22, III: 12, and IV: 25 genes; Figure 1). The HD‐Zip protein content in *S. alopecuroides* was similar to that in soybeans; this is probably because they are both members of the leguminous family.

### 3.2 Chromosome location analysis

**FIGURE 2:**
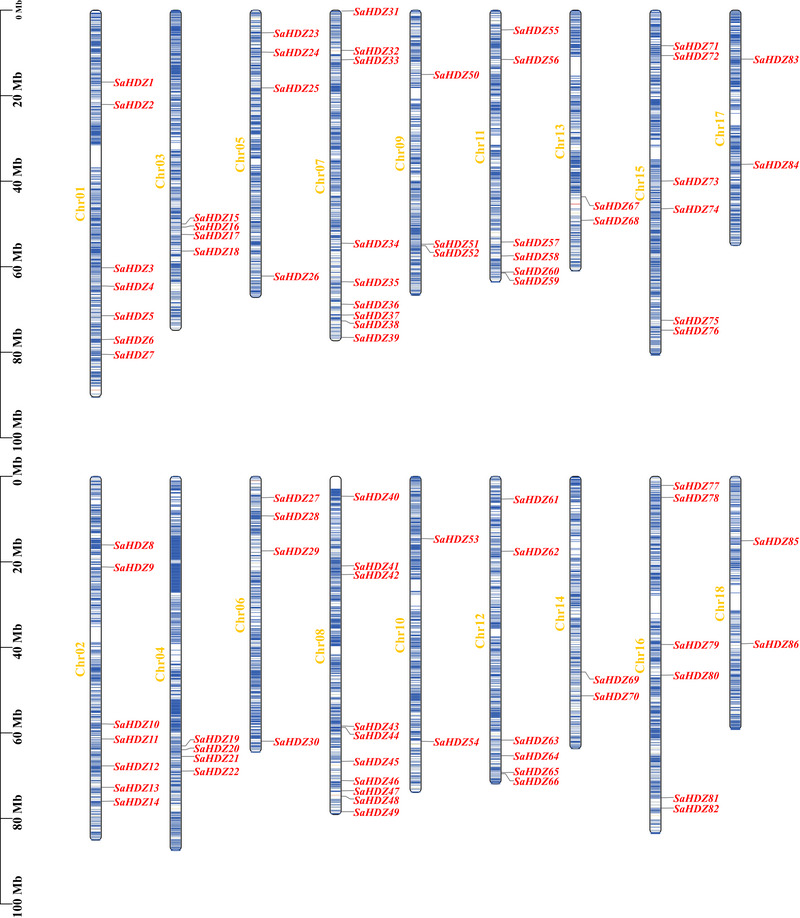
Chromosomal location of *SaHDZ* (*Sophora alopecuroides* HD‐Zip family) genes in *S*. *alopecuroides*. The different colors of the chromosome column represent gene density, which is calculated as the number of genes contained in the chromosome per 100,000 base pairs; dark colors indicate an increased number of genes.

Further analysis of the distribution of *SaHDZ* genes revealed that the 86 identified *SaHDZ* genes were distributed on 18 chromosomes (Figure 2). The highest number of *SaHDZ* genes was on chromosomes 8 and 7, with 10 and 9 *SaHDZ* genes, respectively, followed by seven *SaHDZ* genes each on chromosomes 1 and 2. The lowest number was two *SaHDZ* genes each on chromosomes 10, 13, 14, 17, and 18. The number of *SaHDZ* genes on the other chromosomes ranged from three to six. Analysis of each subfamily showed that 27 members of subfamily I were distributed on chromosomes 3, 4, 5, 6, 7, 8, 13, 14, 15, and 16, whereas 22 members of subfamily II were distributed on 12 chromosomes, including chromosomes 7 and 8. Twelve members of subfamily III were distributed across 10 chromosomes, three of which were found on chromosome 12. The 25 genes of subfamily IV were mainly distributed across 14 chromosomes, including chromosomes 1, 2, 7, 8, 11, 12, 15, and 16, with the widest relative distribution. Further analysis showed that the *SaHDZ* gene subfamilies were alternately distributed on 18 chromosomes in *S. alopecuroides* and that the *SaHDZ* genes on chromosomes 7, 8, 9, and 10 did not correspond completely, which may have been caused by chromosomal variations during the evolution of *S. alopecuroides*.

### 3.3 SaHDZ gene structure and conserved motif analysis

**FIGURE 3:**
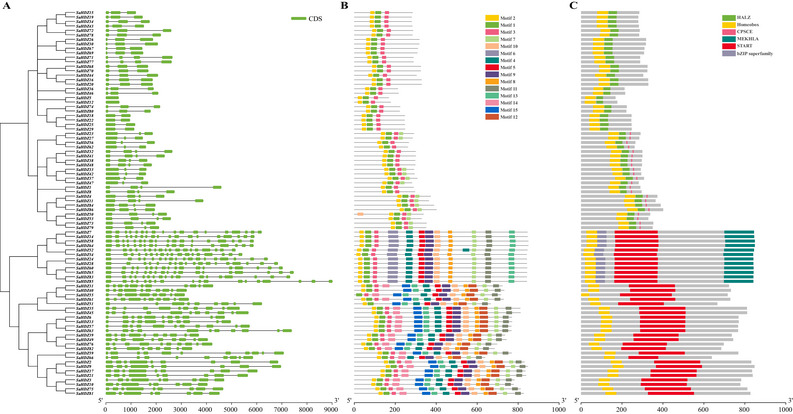
Characterization of *S*. *alopecuroides* HD‐Zip (homologous domain leucine zipper) genes. (A) Gene structure, (B) amino acid motif, and (C) protein conserved domain analyses. *SaHDZ*, *Sophora alopecuroides* HD‐Zip family.

To understand the structural characteristics of *SaHDZ* genes, we analyzed their exons, introns, and conserved motifs. The results showed that the gene structures of the same subfamily were similar, with the gene structures of subclasses I and II containing two to four exons, those of subclass III containing 17–18 exons, and those of subclass IV containing 8–11 exons (Figure 3A). According to our conserved motif analysis, 15 motifs were identified in the amino acid sequence of the SaHDZ protein, among which three motifs were identified in subfamily I, namely, motifs 1, 2, and 3; these corresponded to the conserved domains HALZ and HB (Figure 3B,C).

One additional motif (motif 7) was present in subclass II, and the corresponding conserved domain was CPSCE. Subfamily III contained 13 motifs corresponding to the conserved domains HB, the bZip superfamily, START, and MEKHLA; MEKHLA was unique to members of subfamily III and corresponded to motif 13. Subfamily IV contained 14 motifs corresponding to the conserved domain HB and START. Overall, there were similarities among the various subclasses of the HD‐Zip protein family, but they also possessed distinct characteristics, which may explain the functional differences among the members of the various subfamilies.

### 3.4 Cis‐regulatory element analysis of SaHDZ gene promoters

**FIGURE 4:**
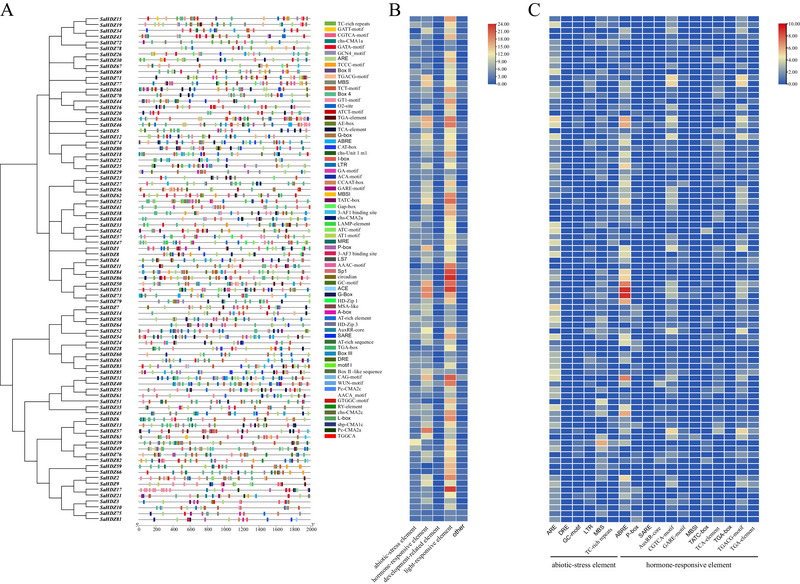
Prediction of cis‐acting elements in *SaHDZ* (*Sophora alopecuroides* HD‐Zip family) gene promoters. (A) The distribution of cis‐acting elements on promoters. (B) Statistics on the number of cis‐acting elements classified. (C) Heat map of cis‐acting element distribution in the broad category of abiotic stress and hormone responses.

To determine the cis‐acting elements of the *SaHDZ* gene promoters, PlantCARE was used to analyze the 2000 bp cis‐acting elements. The results identified 71 cis‐regulatory elements in the promoter regions of the 86 members of the HD‐Zip gene family, and each gene promoter contained 12–27 cis‐acting elements (Figure 4A). Comparison of the predicted cis‐acting elements among the subfamily members revealed no regular differences, thus suggesting that members of the *S. alopecuroides* HD‐Zip family may have a wide range of functions in plants. Among the predicted cis‐acting elements, several HD‐Zip family member promoter regions contained HD‐Zip 1 and HD‐Zip 3, thus suggesting that there may be mutual regulatory effects among the members of the HD‐Zip family (Figure 4A). The identified cis‐acting elements were classified as abiotic stress response, hormone‐responsive, light‐responsive, and development‐related elements (Figure 4B).

Light‐responsive elements were the most abundant, followed by hormone‐responsive and abiotic‐stress elements. We found that the promoter regions of all members of the HD‐Zip family contained similar cis‐acting elements and that the distribution of different types of elements tended to be consistent. This suggests that there may be functional redundancies among members of the *SaHDZ* genes.

To elucidate the role of SaHDZ in abiotic stress, we counted the number of cis‐acting elements involved in the abiotic stress and hormone responses. The results showed that ARE and MBS were the elements most involved in abiotic stress, whereas ABA responsive element (ABRE), the CGTCA‐motif, and the TGACG‐motif were the most involved in hormone response (Figure 4C).

### 3.5 Expression patterns of *SaHDZ* genes based on RNA‐sequencing analysis

**FIGURE 5:**
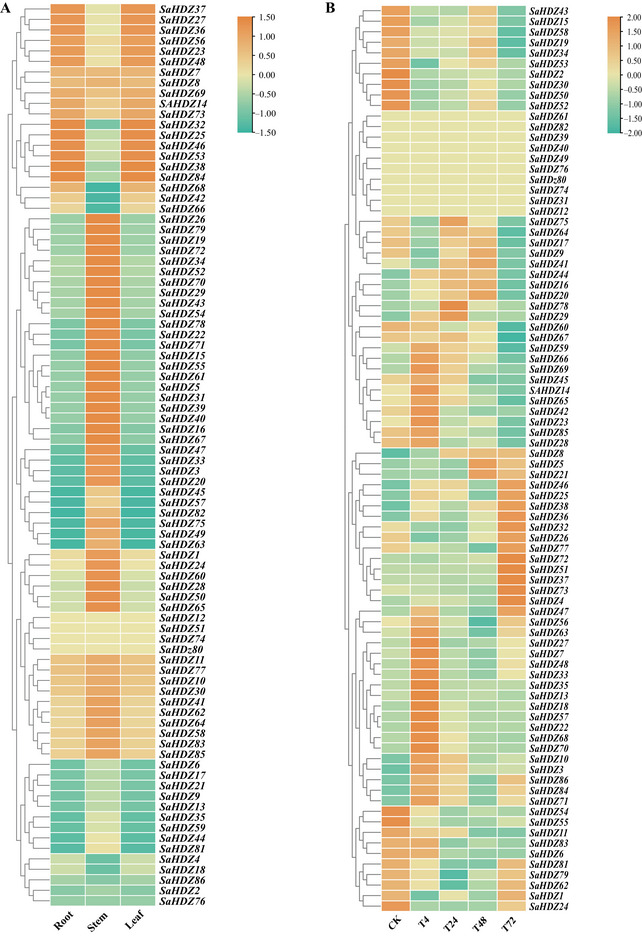
Heat map of *SaHDZ* (*Sophora alopecuroides* HD‐Zip family) gene expression pattern in *S*. *alopecuroides*. (A) The expression levels of SaHDZ in different tissues. (B) The expression levels of SaHDZ at different periods of salt stress.

The expression levels of *SaHDZ* in different tissues were analyzed according to the transcriptome sequencing results for different *S. alopecuroides* tissues (roots, stems, and leaves). The results showed that the expression levels of *SaHDZ15/16/19/20/34/43/67/70/71* (HD‐Zip I), *SaHDZ33/47/62* (HD‐Zip II), and *SaHDZ24/28/52/54/58* (HD‐Zip III) were the highest in the stems, those of *SaHDZ23/37/38/84* (HD‐Zip II) were the highest in the roots, and those of *SaHDZ2/9/17/21* (HD‐Zip IV) were the highest in the leaves (Figure 5A). Further analysis showed that subfamilies II and III were mainly expressed in the roots and stems, while subfamily IV was mainly expressed in the leaves. This may be due to functional differences among the different HD‐Zip subfamily genes in *S. alopecuroides*.

**FIGURE 6:**
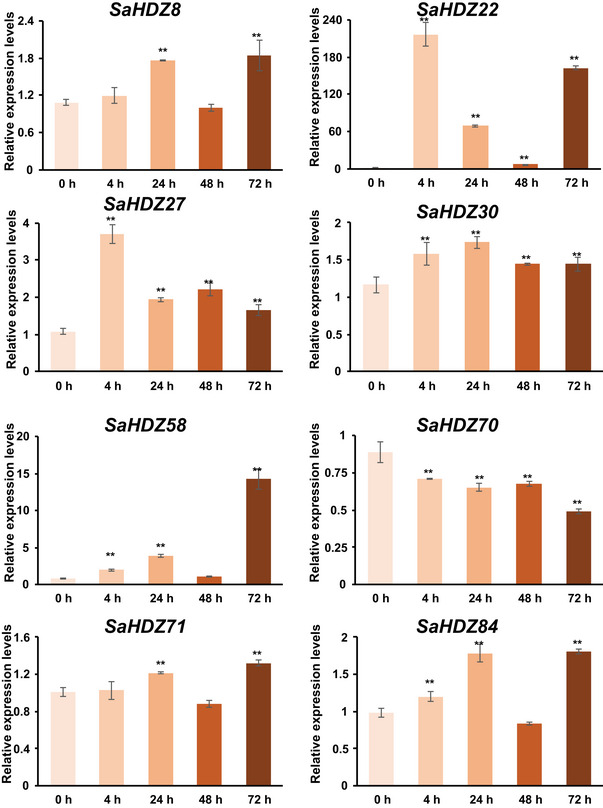
Relative expression levels of *SaHDZ* (*Sophora alopecuroides* HD‐Zip family) in *S*. *alopecuroides* at different periods under salt stress. Data represent the means ± SD, with three biological replicates. Significance was calculated via one‐way analysis of variance (ANOVA) using GraphPad. *p < 0.05; **p < 0.01.

The expression levels of *SaHDZ* genes were analyzed after several periods of salt stress in *S. alopecuroides* (Figure 5B). The results showed that the expression levels of *SaHDZ18/22/46/68/70/78* (HD‐Zip I) and *SaHDZ84/86* (HD‐Zip II) were significantly increased after salt stress. *SaHDZ31/39/40/49/61/76/82* (HD‐Zip IV) did not change significantly under salt stress, while *SaHDZ2/30/50/52* (HD‐Zip I‐IV) were significantly lower. These results indicated that subclass I and II members of *S. alopecuroides* showed positive response to salt stress, while subfamily IV members showed no significant difference. Analysis of the expression trend of each member under salt stress showed that *SaHDZ18/22* (HD‐Zip I), *SaHDZ23* (HD‐Zip II), *SaHDZ28/85* (HD‐Zip III), and *SaHDZ10/13/57/59/66* (HD‐Zip IV) were first upregulated and then downregulated after salt stress; *SaHDZ15/19/34/43* (HD‐Zip I), *SaHDZ53* (HD‐Zip II), *SaHDZ58* (HD‐Zip III), and *SaHDZ9/17* (HD‐Zip IV) were first downregulated and then upregulated; and *SaHDZ8* (HD‐Zip II) was consistently upregulated. qRT‐PCR analysis results indicate that SaHDZ8/22/27/30/58 was significantly upregulated under salt stress (Figure 6), which is consistent with the results of the transcriptome analysis (Figure 5B). These results suggest that members of the *S. alopecuroides* HD‐Zip family exhibit a response to salt stress

### 3.6 Overexpression of SaHDZ22 improves the salt tolerance of *Arabidopsis* during the germination stage

**FIGURE 7:**
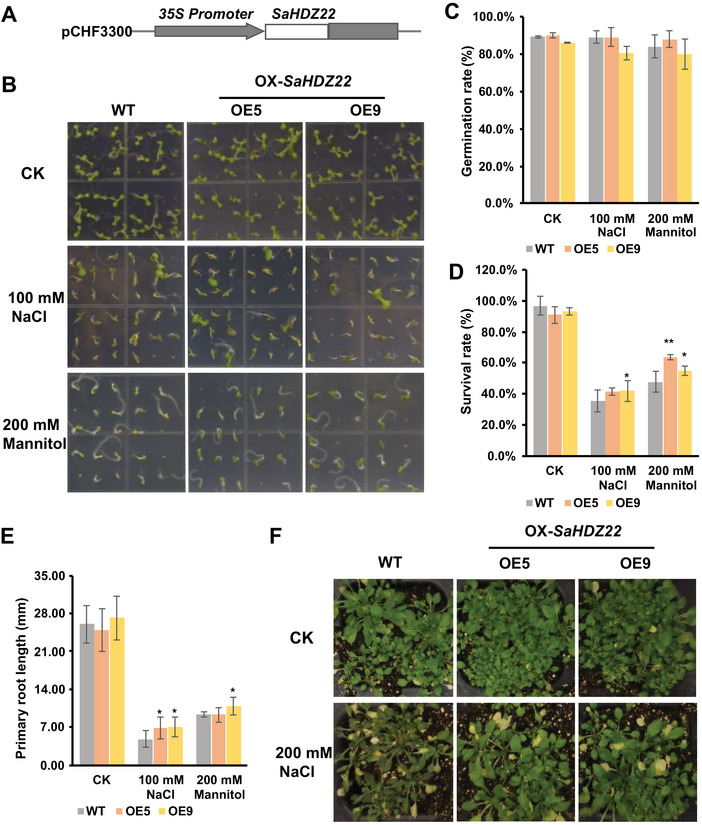
Analysis of salt tolerance in *Arabidopsis thaliana* overexpressing *SaHDZ22*. (A) Plant expression vector recombination model. (B) Germination of seeds under salt stress and osmotic stress treatment. (C) Statistics of germination rate. (D) Statistics of survival rate. (E) Primary root length statistics. (F) Salt stress treatment at seedling stage. CK, control; WT, wild type; OE5 and OE9: *SaHDZ22* overexpression strains. Significant difference at *p < 0.05 and **p < 0.01. *SaHDZ*, *Sophora alopecuroides* HD‐Zip family.

To verify the role of SaHDZ22 in plant salt tolerance, we overexpressed SaHDZ22 in the model plant *Arabidopsis* by genetic transformation (Figure 7A). Salinity tolerance analysis at the germination stage showed that there was no difference in germination rate and survival rate between the overexpression lines (OE5 and OE9) and WT under the control condition (Figure 7B,C). There was no significant difference in germination rate between OE5, OE9, and WT under 100 mM NaCl treatment (Figure 7B,C). However, the survival rate of OE9 was significantly higher than that of WT under salt stress, and the taproot lengths of OE5 and OE9 were significantly higher than that of WT (Figure 7D,E). These results suggest that overexpression of SaHDZ22 enhances the tolerance of *Arabidopsis* to salt stress.

OE5, OE9, and WT seedlings were treated with mannitol to simulate osmotic stress (Figure 7D,E). The results showed that the survival rate of OE5 and OE9 seedlings was significantly higher than that of WT under osmotic stress, and the taproot length of OE9 seedlings was significantly longer than that of WT. These results suggest that the overexpression of *SaHDZ22* may regulate the tolerance to salt stress by regulating osmotic stress in *Arabidopsis*. Salt tolerance analysis at the seedling stage showed that OE5 and OE9 grew significantly better than WT under 200 mM NaCl treatment (Figure 7F), indicating that *SaHDZ22* played a positive role in *Arabidopsis* salt tolerance.

### 3.7 Interacting protein predictions

**FIGURE 8:**
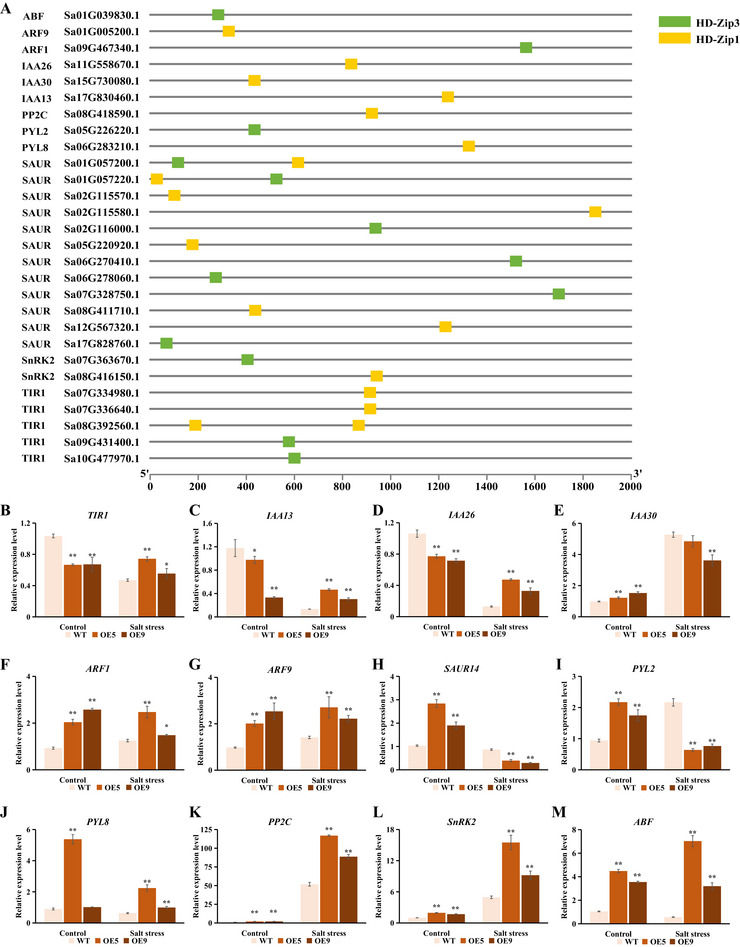
Analysis of HD‐Zip (homologous domain leucine zipper) binding elements in auxin (AUX) and abscisic acid (ABA) signaling pathway gene promoters and analysis of expression levels in *Arabidopsis*. (A) Binding sites of promoter regions of HD‐Zip 1 and HD‐Zip 3. (B–M) Relative expression levels of genes in overexpressed strain of *SaHDZ22* (OE) and wild type (WT) plants under control and salt stress conditions. ABF, ABA responsive element binding factor; ARF, auxin response factor; IAA, auxin‐responsive protein IAA; PP2C, protein phosphatase 2C; PYL, abscisic acid receptor PYR/PYL family; SAUR, SAUR family protein; SnRK2, *Abrus precatorius* serine/threonine‐protein kinase SAPK2‐like; TIR1, transport inhibitor response 1. Data represent the mean ± SD, with three biological replicates. Significance was calculated using one‐way analysis of variance (ANOVA) via GraphPad. *p < 0.05; **p < 0.01.

In this study, 67 interacting proteins were predicted based on the identification of HD‐Zip proteins in *S. alopecuroides*, among which 19 belonged to the HD‐Zip family, corresponding to 64 SaHDZ proteins (18 proteins in subfamily I, 20 in II, 8 in III, and 18 in IV; Figure 8). In total, 412 pairs of 67 interacting proteins interacted with each other, and 46 pairs of interacting proteins were identified. Among the 19 HD‐Zip protein families, 36 pairs of proteins interacted with each other, 10 of which were known to interact. Interactions between the HD‐Zip I subfamily proteins SaHDZ26/30, SaHDZ18/22, SaHDZ68/70, SaHDZ67/69, and SaHDZ25/29 were identified. Additionally, interactions among SaHDZ1/8, SaHDZ4/11, SaHDZ23/27, and SaHDZ32/41 of the HD‐Zip II subfamily proteins were identified (Figure S1).

The results of the SaHDZ22 protein interaction prediction showed that the homologous protein ATHB‐12 (found in *A. thaliana*) had the highest similarity with SaHDZ22. A total of 29 proteins were predicted to interact with each other, 14 of which had a “combined score” >0.4 (Figure 8). SaHDZ22 interacted with CBL (calcineurin B‐like)‐interacting serine/threonine‐protein kinase 11, HB protein ATH1, HB‐LZ zipper protein ATHB‐5, and probable protein phosphatase 2C 78 (SAG113). After binding to ATHB‐12, the predicted interacting proteins of SaHDZ22 included ABA signaling pathway‐related proteins, such as ABF3 and ABI1. Other proteins that interact with SaHDZ22 included AT‐hook motif nuclear‐localized protein 12, HB‐LZ zipper protein MERISTEM L1, GATA transcription factor (GATA2 and GATA9), G‐box‐binding factor 1, NAC domain‐containing protein 72, sugar transport protein 14, and serine/threonine‐protein kinase WAG1 (Table S4).

### 3.8 HD‐Zip family transcription factors regulate AUX and ABA signaling pathways

Promoter sequence analysis of AUX and ABA signal transduction pathway genes in *S. alopecuroides* indicated that HD‐Zip 1 and HD‐Zip 3 binding sequences were involved upstream of 28 genes, including *TIR1*, *IAA*, *ARF*, *SAUR*, *PYL*, *PP2C*, *SnRK2*, and *ABF* (Figure 8A). These results suggest that HD‐Zip family transcription factors in *S. alopecuroides* may be involved in regulating the AUX and ABA signaling pathways. The expression levels of AUX and ABA signal transduction pathway genes in transgenic *A*. *thaliana* were quantitatively analyzed (Figure 8B–M). The results showed that under normal conditions, *TIR1*, *IAA13*, and *IAA26* expression levels in OE plants were downregulated compared with those in WT, but were upregulated compared with those in the WT under salt stress. The *IAA30*, *SAUR14*, and *PYL2* expression levels in the OE strains were higher than those in the WT under control conditions, but were significantly higher than those in WT under salt stress. Simultaneously, the *ARF1*, *ARF9*, *PYL8*, *PP2C*, *SnRK2*, and *ABF* expression levels in OE lines were significantly higher than those in WT under control and salt stress conditions. Notably, there were complementary changes in the expression levels of AUX signaling pathway genes under control and salt stress conditions, suggesting that SaHDZ22 may be involved in regulating plant growth in *A*. *thaliana* by regulating AUX signaling pathway genes. The expression levels of ABA signaling pathway genes in OE strains were significantly higher than those in WT under both control and salt stress conditions, suggesting that *SaHDZ22* may have a positive regulatory effect on ABA signaling pathway. These results suggest that *SaHDZ22* may be involved in salt stress responses in *A*. *thaliana* by regulating the expression of AUX and ABA signaling pathway genes, which provides a reference for further exploring the mechanism of HD‐Zip family transcription factors in plant salt tolerance.

### 3.9 HD‐Zip transcription factors may respond to salt stress by coordinating the balance of AUX and ABA signal transduction

**FIGURE 9:**
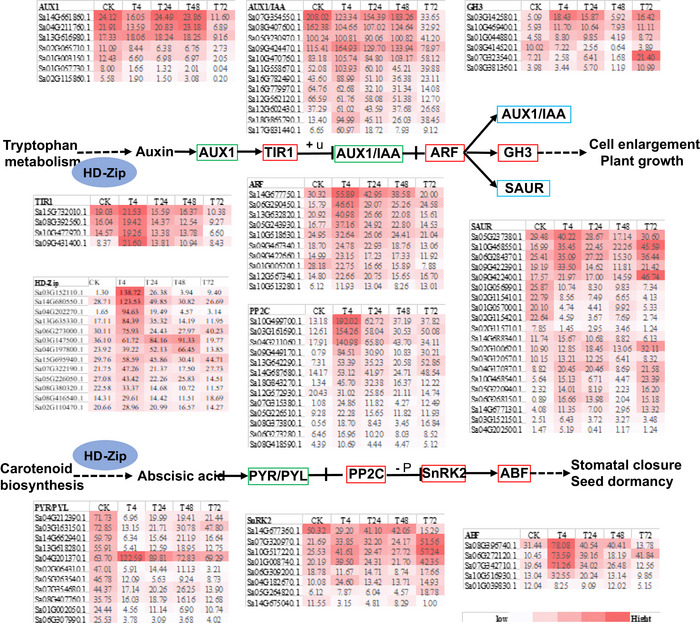
Overview of the relationship between differentially expressed genes in the auxin and abscisic acid signaling pathway of *S. alopecuroides* under salt stress. Values are average transcripts per kilobase per million mapped reads (TPM) values of each sample in each group. ABF, ABA responsive element binding factor; ARF, auxin response factor; AUX1, auxin influx carrier (AUX1 LAX family); GH3, auxin responsive GH3 gene family; HD‐Zip, homologous domain leucine zipper; IAA, auxin‐responsive protein IAA; PP2C, protein phosphatase 2C; PYR/PYL, abscisic acid receptor PYR/PYL family; SAUR, SAUR family protein; SnRK2, *Abrus precatorius* serine/threonine‐protein kinase SAPK2‐like; TIR1, transport inhibitor response 1.

Through the SaHDZ protein interaction network, we found that SaHDZ may be involved in regulating AUX and ABA signal transduction pathways. Therefore, based on the transcriptome, we analyzed the expression trends of AUX and ABA signal transduction pathways in response to salt stress (Figure 9). In the AUX signaling pathway, TIR1, ARF, and GH3 expression were consistent with HD‐Zip family genes, while AUX1, IAA, and SAUR were mainly downregulated after salt stress. This downregulation may be due to *S. alopecuroides* improving stress resistance by slowing down growth under salt stress. In the ABA signal transduction pathway, PYR/PYL was significantly downregulated under salt stress, while PP2C and ABF were upregulated and then downregulated, which was consistent with the expression trend of HD‐Zip transcription factors. Among them, ABI1 (SA06G273280.1) and ABF3 (SA08G396740.01), which interacted with SaHDZ22 (SA04G20227.1), were significantly upregulated after salt stress. In addition, SnRK2 was upregulated, downregulated, and then upregulated under salt stress. These results suggest that HD‐Zip transcription factors may respond to salt stress by coordinating the balance of AUX and ABA signals.

### 3.10 SaHDZ22 locates to the nucleus and its C‐terminal has self‐activating activity

**FIGURE 10:**
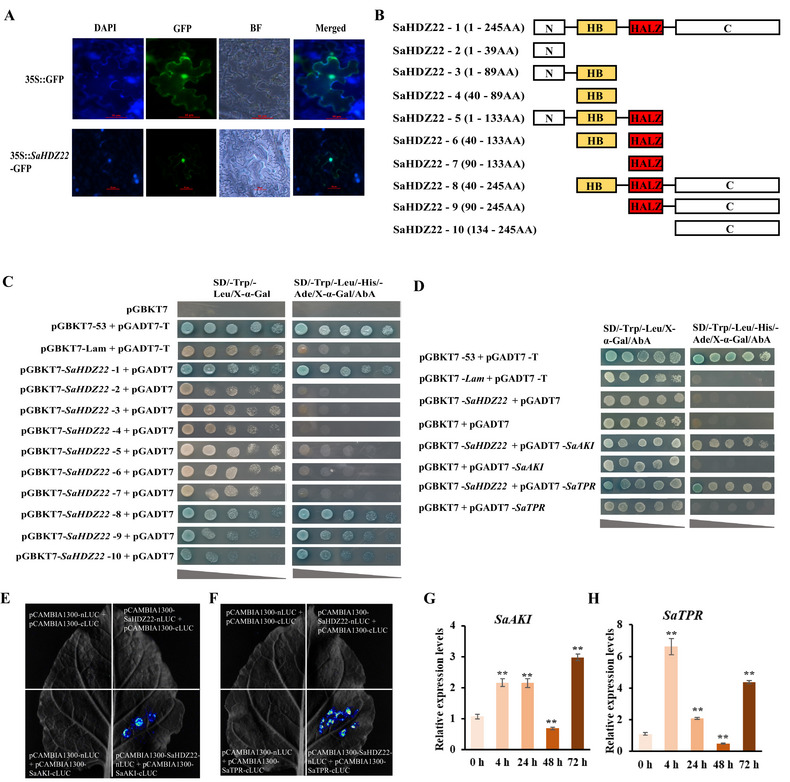
Subcellular mapping of SaHDZ22 and interaction analysis of SaHDZ22 with *Sophora alopecuroides* adenylate kinase protein (SaAKI) and SaTPR. (A) Transient expression of SaHDZ22 in the lower epidermis of tobacco. (B) The amino acid sequence corresponding to the construction reaction. (C) Yeast color reaction. (D) Yeast two‐hybridization verification of interactions between SaHDZ22 with SaAKI or SaTPR. (E and F) Luciferase verification of interactions between SaHDZ22 with SaAKI or SaTPR. (G and H) Relative expression levels of SaAKI and SaTPR in *S*. *alopecuroides* under salt stress. Data represent the means ± SD, with three biological replicates. Significance was calculated via one‐way analysis of variance (ANOVA) using GraphPad. *p < 0.05; **p < 0.01.

Prediction of the subcellular localization of SaHDZ proteins in *S. alopecuroides* indicated that all genes were localized in the nucleus, which is also a characteristic of HD‐Zip family proteins as transcription factors. In this study, transient expression of SaHDZ22 in the lower epidermal cells of tobacco leaves was observed after Hoechst staining, and the results showed that green fluorescence from SaHDZ22 fused with GFP overlapped with the nucleus, thus indicating that SaHDZ22 was localized in the nucleus (Figure 10A).

The SaHDZ22 gene was inserted into the pGBKT7 vector for self‐activation capacity studies, and the results showed that SaHDZ22 was capable of self‐activation. To further analyze the self‐activated fragments produced by SaHDZ22, we truncated SaHDZ22 and constructed 10 reactions for self‐activation verification. The results showed that the C‐terminus of SaHDZ22 had self‐activating activity (Figure 10B,C), and the conserved domain of the SaHDZ22 gene had no self‐activating activity. This allowed us to further screen for proteins that interact with SaHDZ22.

### 3.11 Screening and verification of SaHDZ2‐interacting proteins

Truncated SaHDZ22 was hybridized with a yeast nuclear library to screen for interacting proteins. A total of 66 monoclones were selected from the SD/‐Trp/‐Leu/‐His/‐Ade/X‐α‐Gal/AbA screening medium and grown in the SD/‐Trp/‐Leu/‐His/‐Ade/X‐α‐Gal/AbA medium. Among them, 56 (84.8%) of the monoclones were capable of growth (Figure S2A), 23 turned blue, and yeast liquid PCR detection was performed. Of these, 13 were selected for plasmid extraction, *Escherichia coli* transformation, and sequencing (Figure S2B). Sequence alignment and conserved domain analysis were performed for glutamate‐pyruvate transaminase (GTP) cyclic hydrolase II, Y‐family DNA polymerase, SRC2‐like, adenylate kinase homologous enzyme, and tetra tripeptide repeat (TPR) superfamily proteins (Table S5).

The SaAKI and SaTPR proteins obtained were amplified using a plasmid, and the co‐transmutation yeast two‐hybrid system was used for verification. The results showed that SaAKI and SaTPR could grow on the SD/‐Trp/‐Leu/‐His/‐Ade/X‐α‐Gal/AbA medium after co‐transmutation with SaHDZ22 and were capable of turning blue, while the AD and BD plasmid of SaAKI and SaTPR could not grow on the SD/‐Trp/‐Leu/‐His/‐Ade/X‐α‐Gal/AbA medium (Figure 10D). The interactions of SaHDZ22 with SaAKI and SaTPR were verified using complementary luciferase assays. The results showed that SaHDZ22 interacted with SaAKI and SaTPR to activate LUC expression in plants (Figure 10E,F). To our knowledge, this is the first study to demonstrate that HD‐Zip family proteins can interact with adenylate kinase homologues and TPR superfamily proteins.

The expression level analysis showed that *SaAKI* and *SaTPR* were first upregulated and then downregulated under salt stress, and then upregulated again at 72 h (Figure 10G,H). This expression trend was fully consistent with that of *SaHDZ22*, which further indicated that SaHDZ22 may be involved in regulating the response of *S. alopecuroides* to salt stress by co‐acting with SaAKI and SaTPR.

## 4 DISCUSSION

HD‐Zip transcription factor family genes have been identified in many plants. The number of HD‐Zip transcription factors reported differs according to species, including 88 in soybeans (Chen et al., 2014), 47 in foxtail millet (*Setaria italica* L.) (Chai et al., 2018), 83 in radishes (K. Wang, Xu, et al., 2022), 43 in potatoes (*Solanum tuberosum* L.) (W. Li, Dong, et al., 2019), 45 in sesame (*Sesamum indicum* L.) (Wei et al., 2019), 40 in cucumbers (*Cucumis sativus* L.) (Sharif et al., 2020), 33 in tea plants (*Camellia sinensis* L.) (Shen et al., 2019), and 32 in Japanese apricots (*Prunus mume* L.) (L. Li, Zheng, et al., 2019). The identification of HD‐Zip family proteins in most of these plants was based on results from the model plant *A. thaliana*. With continuing advances in whole‐genome sequencing technology, an in‐depth understanding of plant genomes will further promote our understanding of the HD‐Zip family of proteins. In this study, we identified 86 *SaHDZ* genes, which is similar to the number found in soybeans. Phylogenetic tree analysis showed that the number of HD‐Zip subfamily III genes identified in *S. alopecuroides* and soybean was the smallest, whereas the numbers of subfamilies I and IV were relatively high. Gene structure analysis showed that *SaHDZ* and soybean HD‐Zip family genes were similar, and amino acid sequence analysis revealed high motif similarity, which further indicated the reliability of the identified HD‐Zip transcription factor genes in *S. alopecuroides*. Chromosome distribution analysis revealed that the 86 *SaHDZ* genes were distributed on 18 chromosomes, whereas the 88 HD‐Zip genes in soybean are distributed on 20 chromosomes (Chen et al., 2014). In *Ophiorrhiza pumila*, 32 HD‐Zip genes are distributed on 10 chromosomes (J. Wang et al., 2023), and 40 are distributed on seven chromosomes in cucumbers (*C. sativus* L.) (Sharif et al., 2020). This indicates that HD‐Zip transcription factor family genes, which are unique to plants, are distributed across many chromosomes, which may be related to the diversity of their functions.

The function of HD‐Zip transcription factors in plant resistance to various stresses (including salt, alkali, drought, high temperatures, and low temperatures) has been reported (Y. Li et al., 2022); therefore, it is important to explore their role in the underlying mechanism of plant salt tolerance. HD‐Zip transcription factors regulate salt tolerance via the ABA signaling pathway (Y. Li, Yang, et al., 2022). *GhHB1* (HD‐Zip I) expression in cotton is strongly induced by ABA and NaCl (Ni et al., 2008). *ZmHDZ1* and *ZmHDZ10* in maize participate in the regulation of the salt stress responses via the ABA signaling pathway, in which *ZmHDZ1* negatively regulates salt tolerance, while *ZmHDZ10* positively regulates the salt stress response by regulating the expression levels of *P5CS1*, *RD29B*, *ABI1*, and *RD22* (Q. Wang et al., 2017; Y. Zhao et al., 2014). Overexpression of the HD‐Zip I gene *MsHB7* can regulate the expression of *MsSnRK2.6* and *MsABI1*, which can regulate salt tolerance in alfalfa (X. Li, Hou, et al., 2022). CaHAT1, a target protein of CaSnRK2.6, was enhanced under ABA treatment and drought stress in *Capsicum annuum* (Baek et al., 2023). In this study, 40 of the 86 *SaHDZ* genes were strongly induced by salt stress, with the expression levels of six subclass I genes being significantly upregulated. In addition, expression levels of 28 *SaHDZ* genes were first downregulated and then upregulated after salt stress, which may be due to the response times of different *HD‐Zip* genes to salt stress. This result may also be due to the role of HD‐Zip family genes in maintaining energy balance during plant responses to salt stress. Prediction of the proteins that interact with SaHDZ revealed SaHDZ18/22/25/29/67/69 may interact with the ABA signaling pathways ABF3, ABI, and SAG113, further suggesting that HD‐Zip proteins in *S. alopecuroides* may be involved in regulating the salt stress response via ABA signaling pathways. HD‐Zip proteins function via the AUX and ethylene signaling pathways. Upon interfering with the expression of *VAHOX1* (a HD‐Zip I subfamily member) in tomatoes using RNAi, accelerated ripening, enhanced sensitivity to ethylene, and increased total carotene content and ethylene production were observed (F. Li, Fu, et al., 2022). Altering the expression of VAHOX1 affects the accumulation of transcripts of various genes involved in ethylene biosynthesis, signal transduction, and cell wall modification (F. Li, Fu, et al., 2022). In this study, we predicted that the AP2‐like ethylene‐responsive transcription factor AIL6 interacts with SaHDZ24/28 and SaHDZ52/54. The HD‐Zip transcription factor SlHB15A in tomatoes decreases the levels of jasmonate‐isoleucine (JA‐Ile) by inhibiting *the expression of SlJAR1*, a gene involved in JA‐Ile biosynthesis that regulates shedding (X. Liu et al., 2022). SlHB15A mediates antagonism between AUX and JA‐Ile during tomato pedicle shedding, whereas AUX inhibits shedding through the SLHB15A‐SLJAR1 module (X. Liu et al., 2022). Multiple HD‐Zip proteins in *S. alopecuroides* can interact with AUX efflux carrier component 3. The HD‐Zip II family transcription factors HAT3 and ATHB4 play important roles in establishing the radial symmetry pattern of female *Arabidopsis* by coordinating plant–hormone interactions (Carabelli et al., 2021). We found that the promoters of *PYL*, *PP2C*, *SnRK2*, and *ABF*, as well as key genes of the AUX signaling pathway (*TIR1*, *IAA*, *ARF*, *SAUR*) and ABA signaling pathways in *S. alopecuroides*, contain HD‐Zip1 and HD‐Zip 3 acting elements. Using expression dynamics of these genes under salt stress, we hypothesized that HD‐Zip family transcription factors may play a role in regulating the balance between growth and stress resistance during plant salt tolerance.

HD‐Zip transcription factor proteins participate in the response to salt stress by regulating compatible solute concentrations and clearing ROS. Overexpression of *Arabidopsis AtHDG11* in cotton can significantly reduce malondialdehyde (MDA) levels under salt and drought stress, while increasing the activities of superoxide dismutase (SOD) and catalase (CAT), thereby protecting plants from oxidative damage and improving their salt and drought tolerance by removing ROS (L. H. Yu et al., 2016). Overexpression of the physic nut HD‐Zip gene *JcHDZ07* reduced the proline content and CAT and SOD activities in *A. thaliana* and rice under salt stress, indicating that JcHDZ07 negatively regulates salt stress responses (Tang, Bao, et al., 2019; Tang, Wang, et al., 2019). After overexpression of MdHB7‐like protein, the MDA content and relative electrolyte leakage of apple leaves were lower than those of the wild type, and the Na^+^/K^+^ ratios in the roots, stems, and leaves were also significantly lower than those in the wild type (S. Zhao et al., 2021). Overexpression of the pepper HD‐Zip II gene *CaHB1* in tomatoes improved salt tolerance and enhanced the photosynthetic capacity of transgenic lines (Oh et al., 2013). In this study, overexpression of *SaHDZ22* improved *Arabidopsis* tolerance to salt and osmotic stress. Our SaHDZ protein interaction network suggested that SaHDZ‐interacting proteins include amino acid kinases, diacylglycerol kinases, and E3 ubiquitin‐protein ligases, which may be key to the function of SaHDZ.

HD‐Zip plays an important role in the synthesis of lignin and anthocyanins and may play a positive role in abiotic stress responses. Overexpression of *MeHDZ14* in *Manihot esculenta* Crantz (transgenic cassava) affected hormone and lignin synthesis and changed the morphology of internodal cells, thus resulting in significantly shortened internodal spacing, plant dwarfization, and contraction of the lower epidermal cells of leaves, thereby causing the front of the cassava leaves to curl (X. Yu et al., 2023). MeHDZ14 can directly regulate several drought stress response genes, including those that regulate lignin synthesis and cell wall development, such as caffeic acid 3‐O‐methyltransferase (X. Yu et al., 2023). This suggests that *MeHDZ14* regulates cassava plant development and drought stress responses by inhibiting gibberellin and lignin synthesis (X. Yu et al., 2023). The *Arabidopsis* transcription factor GLABRA2 (GL2) is a transcription suppressor that inhibits anthocyanin biosynthesis by binding to the anthocyanin activator AtPAP2 and AtMYB113 promoter to inhibit their expression (LaFountain & Yuan, 2021). Under drought stress, this HD‐Zip II transcription factor binds and deactivates the MYB‐bHLH‐WD40 (MBW) complex, subsequently inhibiting anthocyanin synthesis. However, under ABA, binding of the HD‐Zip II transcription factor is disrupted, and Myb75 plays a role in promoting anthocyanin synthesis (Zheng et al., 2019). SaHDZ59/66 has the highest homology with GL2 and interacts with SaHDZ32/41, SaHDZ31/40, mitogen‐activated protein kinase 3, and GATA transcription factors in *S. alopecuroides*. These results provide a basis for further investigations into the function of the *SaHDZ* genes in *S. alopecuroides*.

HD‐Zip proteins are transcription factors, and members of this family are mainly localized in the nucleus. In this study, localization predictions for the 86 identified SaHDZ proteins showed that they were all located in the nucleus. To date, AtHB12 (Shin et al., 2004) and HAT22/ABIG1 (T. Liu et al., 2016) in *Arabidopsis*, OsHOX12 and OsHOX14 in rice (*Oryza sativa*) (Shao et al., 2018), and CaATHB‐12 (R. X. Zhang et al., 2020) and CaHB1 (Oh et al., 2013) in cayenne pepper have been reported to be nuclear proteins. Our results showed that SaHDZ22 was localized in the nucleus, suggesting that it regulates the expression of target genes by binding to the cis‐acting elements of their promoters. These cis‐acting components were predicted to contain HD‐Zip 1 in SaHDZ1/8/13/33/37/47/77 and HD‐Zip 3 in SaHDZ4/21/68/70. This indicates that there are both protein‐interacting relationships among the HD‐Zip protein family members as well as transcription factor–target gene relationships. Some HD‐Zip transcription factors, including ATHB17 in *Arabidopsis* (P. Zhao et al., 2017), MdHB1 in apples (Jiang et al., 2017), and CsGL2‐LIKE in cucumber (Cai et al., 2020), have been identified. This indicates that the localization of HD‐Zip proteins in plant cells is relatively consistent but may change owing to changes in the environment and growth stage. We aim to verify the localization of each member of the HD‐Zip family in future studies.

The yeast two‐hybrid library screening for the SaHDZ22 protein is crucial for discovery of new interacting proteins. As a typical stress‐resistant plant, it is very important to explore new stress‐resistant genes and mechanisms. Self‐activating activity verification showed that the C‐terminal of SaHDZ22 had self‐activating activity, similar to the C‐terminal self‐activating activity of GmHDZ20 in soybean (H. Yang et al., 2021), PsnHDZ63 in poplar (Guo et al., 2021), CsHB5 in citrus (Y. Zhang et al., 2021), and MdHB7‐like in apple (S. Zhao et al., 2021) in previous reports, which all belong to the HD‐Zip I subfamily. This shows that our results are real and reliable. By analyzing the yeast library screening results, we found that the adenylate kinase proteins SaAKI and SaTPR can interact with SaHDZ22, which was verified by the yeast two‐hybrid system and complementary luciferase experiment. Adenylate kinases exist widely in organisms and play important roles in cellular energy homeostasis (Dzheia et al., 1986; L. Yang et al., 2021). In plants, adenylate kinases play important roles in plant growth and development regulation and environmental stress adaptions (L. Yang et al., 2021). Adenylate kinase catalyzes reversible transphosphorylation and is a key enzyme in the maintenance of biological energy metabolism (Pradet & Raymond, 1983). T‐DNA insertion mutants of the *Arabidopsis* adenylate kinase gene, *At2g37250*, increased amino acid levels and enhanced root growth (Carrari et al., 2005). Adenylate kinase (SSN‐U232826) expression in tomatoes is strongly induced by drought stress (Gong et al., 2010). SlADK10, another adenylate kinase in tomatoes, plays a positive regulatory role in drought response, and *SlADK* is responsive to most plant hormone treatments (including ABA, ETH, etc.) (L. Yang et al., 2021). Meanwhile, *StADK* gene expression in potatoes changes significantly under salt, drought, ABA, cold, and H_2_O_2_ treatments, indicating that *StADK* actively participated in abiotic stress responses (X. Li et al., 2023). Adenylate kinase can maintain mitochondrial adenosine triphosphate (ATP) synthesis during water stress, which is essential for maintaining plastid function, and increased adenylate kinase gene expression can provide additional ATP to maintain cell activity under drought stress (Atkin & Macherel, 2009; Gong et al., 2010). The results of this study confirmed that the adenylate kinase protein, SaAKI, interacts with SaHDZ22, suggesting that SaHDZ22 may be involved in regulating plant cell energy metabolism in response to environmental stress. Interestingly, adenylate kinase (F14F18.170) protein was also found in the protein interaction prediction, which provided a basis for further analysis of the function of SaHDZ22. Tetrapeptide‐like repeat superfamilies with TPR conserved domains are widely involved in plant growth, development, and abiotic stress responses (Rosado et al., 2006). Tetratricopeptide‐like repeat thioredoxin‐like 1 (TTL1) is a positive regulator of ABA signaling during germination and seedling development under osmotic stress (Rosado et al., 2006). TTL1 mutations reduced tolerance to NaCl and osmotic stress, characterized by reduced root elongation, root meristem disturbance, and impaired osmotic response during germination and seedling development (Rosado et al., 2006). SG1 in *Arabidopsis* encodes chloroplast‐localized proteins containing tetrapeptide repeats (Hu et al., 2014). *sg1* mutations disrupt the expression of genes related to chloroplast development, photosynthesis, and chlorophyll biosynthesis, and mutants exhibit slow neocotyledon greening, indicating its necessity in chloroplast development (Hu et al., 2014). *MdTPR16* overexpression in apples regulate drought‐related gene expression and ROS accumulation, which is a positive regulator of the drought stress response (X. Liu et al., 2024). Tomato miR169 plays an important role in responses to abiotic stress, of which the tetrapeptide repeat protein is a target (Rao et al., 2020). In this study, we found that SaTPR interacts with SaHDZ22, which provides a reference and basis for further exploring the mechanism of SaHDZ22 in response to salt stress.

There were some limitations in this study. Due to the immaturity of the genetic transformation system of *S. alopecuroides*, the function of *SaHDZ* genes has not been further verified. Based on the genetic transformation system of other crops, transgenic technology will likely be developed for alopecia in the near future to provide conditions for studying gene function in *S. alopecuroides*. At the same time, the relationship between interacting proteins needs to be further verified through in vitro and in vivo experiments, and the specific mechanism of action of each *SaHDZ* gene needs to be further explored.

## 5 CONCLUSION

This study is the first to complete the identification of HD‐Zip family genes based on whole genome data, with a total of 86 *SaHDZ* genes identified and divided into four subfamilies through phylogenetic analysis. Chromosome localization results showed that the 86 *SaHDZ* genes were distributed on 18 chromosomes. Protein domain and amino acid conserved motif analyses showed high similarity among HD‐Zip protein subfamilies. *SaHDZ* promoter cis‐acting elements were predicted to be within 71 elements. Analysis of expression patterns showed that SaHDZ family members had a positive response to salt stress. Functional analysis showed that the overexpression of *SaHDZ22* could improve the salt tolerance of *Arabidopsis*. The interaction network of SaHDZ proteins was constructed, and the interaction relationships of 19 SaHDZ proteins were predicted. HD‐Zip family transcription factors regulate the balance between plant growth and stress resistance under salt stress by regulating the expression of AUX and ABA signaling pathway genes, including *TIR1*, *IAA*, *ARF*, *SAUR*, *PYL*, *PP2C*, *SnRK2*, and *ABF*. The interaction protein screening and verification of HD‐Zip I member SaHDZ22 showed that SaAKI and SaTPR could interact with SaHDZ22. The response patterns of *SaAKI* and *SaTPR* to salt stress were consistent with those of *SaHDZ22*. These results provide reference for further analysis of the mechanism of HD‐Zip family gene response to salt stress and provide a new gene resource for future crop resistance breeding.

## AUTHOR CONTRIBUTIONS

**Youcheng Zhu**: Conceptualization; data curation; formal analysis; investigation; resources; software; supervision; visualization; writing—original draft; writing—review and editing. **Di Wang**: Conceptualization; formal analysis; investigation; methodology; resources; software; validation; visualization. **Fan Yan**: Data curation; formal analysis; funding acquisition; investigation; methodology; project administration; software; writing—review and editing. **Le Wang**: Data curation; funding acquisition; software; supervision; visualization; writing—review and editing. **Ying Wang**: Data curation; formal analysis; funding acquisition; investigation; project administration; resources; writing—review and editing. **Jingwen Li**: Investigation; methodology; resources; validation; visualization. **Xuguang Yang**: Data curation; formal analysis; investigation; resources; validation; visualization. **Xu Liu**: Investigation; methodology; validation; visualization. **Ziwei Gao**: Data curation; formal analysis; investigation; methodology; software; validation; visualization. **Yajing Liu**: Conceptualization; data curation; formal analysis; funding acquisition; investigation; project administration; resources; software; writing—review and editing. **Qingyu Wang**: Conceptualization; formal analysis; funding acquisition; investigation; methodology; project administration; resources; software; visualization; writing—original draft; writing—review and editing.

## CONFLICT OF INTEREST STATEMENT

The authors declare no conflicts of interest.

## Supporting information


**Table S1** Amino acid sequence of HD‐Zip family proteins of *S. alopecuroides*, *A. thaliana*, and *Glycine max* (L.).
**Table S2** Primers used for detection and real‐time PCR analyses.
**Table S3** *SaHDZ22* sequence truncation statistics.
**Table S4** Annotation of predictive interacting proteins.
**Table S5** Annotation of the interacting proteins of SaHDZ22 in yeast screening.
**Figure S1** Prediction of SaHDZ interacting protein networks in *S. alopecuroides*.
**Figure S2** Screening of SaHDZ22 interacting proteins. (A) Screening of yeast monoclones; (B) PCR detection and screening of protein genes.

## Data Availability

Data will be made available upon request to the corresponding authors.
