# Supplementary material for: Genome‐wide analysis of HD‐Zip genes in Sophora alopecuroides and their role in salt stress response
Source: Plant Genome. 2024 Aug 28;17(3):e20504. doi: 10.1002/tpg2.20504 (PMC12807318; doi:10.1002/tpg2.20504)
Supplement: Supplementary file 1 [file TPG2-17-e20504-s001.docx]

**Table S1** Amino acid sequence of HD-Zip family proteins of *S. alopecuroides*, *A. thaliana* and *Glycine max* (L.).

| name | ID | name | ID |
| --- | --- | --- | --- |
| Gmhdz1 | Glyma0041s00350 | HAT3 | AT3G60390 |
| Gmhdz2 | Glyma01g04890 | HDG1 | AT3G61150 |
| Gmhdz3 | Glyma01g05230 | ATHB12 | AT3G61890 |
| Gmhdz4 | Glyma01g38390 | ANL2 | AT4G00730 |
| Gmhdz5 | Glyma01g40450 | PDF2 | AT4G04890 |
| Gmhdz6 | Glyma01g41581 | ATHB2 | AT4G16780 |
| Gmhdz7 | Glyma01g45070 | HAT1 | AT4G17460 |
| Gmhdz8 | Glyma02g02290 | HDG4 | AT4G17710 |
| Gmhdz9 | Glyma02g02630 | ATML1 | AT4G21750 |
| Gmhdz10 | Glyma02g06560 | HDG6 | AT4G25530 |
| Gmhdz11 | Glyma02g28860 | ATHB8 | AT4G32880 |
| Gmhdz12 | Glyma03g01860 | ATHB40 | AT4G36740 |
| Gmhdz13 | Glyma03g26701 | HAT22 | AT4G37790 |
| Gmhdz14 | Glyma03g30200 | ATHB16 | AT4G40060 |
| Gmhdz15 | Glyma03g34710 | ATHB51 | AT5G03790 |
| Gmhdz16 | Glyma04g05200 | HAT14 | AT5G06710 |
| Gmhdz17 | Glyma04g09000 | ATHB3 | AT5G15150 |
| Gmhdz18 | Glyma04g34341 | HDG9 | AT5G17320 |
| Gmhdz19 | Glyma04g40960 | HDG5 | AT5G46880 |
| Gmhdz20 | Glyma05g01390 | HAT2 | AT5G47370 |
| Gmhdz21 | Glyma05g04990 | HDG7 | AT5G52170 |
| Gmhdz22 | Glyma05g23150 | ATHB52 | AT5G53980 |
| Gmhdz23 | Glyma05g30000 | REV | AT5G60690 |
| Gmhdz24 | Glyma05g30940 | ATHB5 | AT5G65310 |
| Gmhdz25 | Glyma05g33520 | ATHB53 | AT5G66700 |
| Gmhdz26 | Glyma06g09100 | SaHDZ1 | Sa01G011480 |
| Gmhdz27 | Glyma06g13890 | SaHDZ2 | Sa01G014270 |
| Gmhdz28 | Glyma06g20230 | SaHDZ3 | Sa01G033880 |
| Gmhdz29 | Glyma07g01940 | SaHDZ4 | Sa01G037750 |
| Gmhdz30 | Glyma07g01950 | SaHDZ5 | Sa01G043100 |
| Gmhdz31 | Glyma07g02220 | SaHDZ6 | Sa01G048820 |
| Gmhdz32 | Glyma07g05800 | SaHDZ7 | Sa01G051800 |
| Gmhdz33 | Glyma07g08340 | SaHDZ8 | Sa02G073750 |
| Gmhdz34 | Glyma07g14270 | SaHDZ9 | Sa02G076690 |
| Gmhdz35 | Glyma07g34230 | SaHDZ10 | Sa02G093630 |
| Gmhdz36 | Glyma08g06190 | SaHDZ11 | Sa02G096580 |
| Gmhdz37 | Glyma08g13110 | SaHDZ12 | Sa02G102370 |
| Gmhdz38 | Glyma08g14130 | SaHDZ13 | Sa02G107460 |
| Gmhdz39 | Glyma08g15771 | SaHDZ14 | Sa02G110470 |
| Gmhdz40 | Glyma08g21610 | SaHDZ15 | Sa03G146890 |
| Gmhdz41 | Glyma08g21620 | SaHDZ16 | Sa03G147500 |
| Gmhdz42 | Glyma08g21890 | SaHDZ17 | Sa03G148700 |
| Gmhdz43 | Glyma08g40705 | SaHDZ18 | Sa03G152110 |
| Gmhdz44 | Glyma08g40970 | SaHDZ19 | Sa04G197230 |
| Gmhdz45 | Glyma09g02750 | SaHDZ20 | Sa04G197800 |
| Gmhdz46 | Glyma09g07051 | SaHDZ21 | Sa04G199060 |
| Gmhdz47 | Glyma09g16790 | SaHDZ22 | Sa04G202270 |
| Gmhdz48 | Glyma09g26600 | SaHDZ23 | Sa05G226050 |
| Gmhdz49 | Glyma09g29810 | SaHDZ24 | Sa05G230470 |
| Gmhdz50 | Glyma09g34061 | SaHDZ25 | Sa05G237340 |
| Gmhdz51 | Glyma09g37410 | SaHDZ26 | Sa05G264930 |
| Gmhdz52 | Glyma09g37680 | SaHDZ27 | Sa06G273000 |
| Gmhdz53 | Glyma09g40130 | SaHDZ28 | Sa06G277300 |
| Gmhdz54 | Glyma10g38280 | SaHDZ29 | Sa06G284350 |
| Gmhdz55 | Glyma10g39720 | SaHDZ30 | Sa06G309310 |
| Gmhdz56 | Glyma11g00570 | SaHDZ31 | Sa07G311990 |
| Gmhdz57 | Glyma11g03850 | SaHDZ32 | Sa07G320460 |
| Gmhdz58 | Glyma11g04840 | SaHDZ33 | Sa07G322190 |
| Gmhdz59 | Glyma11g20520 | SaHDZ34 | Sa07G347370 |
| Gmhdz60 | Glyma11g37920 | SaHDZ35 | Sa07G355430 |
| Gmhdz61 | Glyma12g08080 | SaHDZ36 | Sa07G360450 |
| Gmhdz62 | Glyma12g10710 | SaHDZ37 | Sa07G362850 |
| Gmhdz63 | Glyma12g32050 | SaHDZ38 | Sa07G364110 |
| Gmhdz64 | Glyma13g00310 | SaHDZ39 | Sa07G368270 |
| Gmhdz65 | Glyma13g05270 | SaHDZ40 | Sa08G370090 |
| Gmhdz66 | Glyma13g23890 | SaHDZ41 | Sa08G378740 |
| Gmhdz67 | Glyma14g10370 | SaHDZ42 | Sa08G380320 |
| Gmhdz68 | Glyma15g01960 | SaHDZ43 | Sa08G400740 |
| Gmhdz69 | Glyma15g13640 | SaHDZ44 | Sa08G401050 |
| Gmhdz70 | Glyma15g18320 | SaHDZ45 | Sa08G408340 |
| Gmhdz71 | Glyma15g42380 | SaHDZ46 | Sa08G412960 |
| Gmhdz72 | Glyma16g02390 | SaHDZ47 | Sa08G415340 |
| Gmhdz73 | Glyma16g34350 | SaHDZ48 | Sa08G416540 |
| Gmhdz74 | Glyma17g06380 | SaHDZ49 | Sa08G420350 |
| Gmhdz75 | Glyma17g10490 | SaHDZ50 | Sa09G432670 |
| Gmhdz76 | Glyma17g15380 | SaHDZ51 | Sa09G455190 |
| Gmhdz77 | Glyma17g16930 | SaHDZ52 | Sa09G455420 |
| Gmhdz78 | Glyma18g01830 | SaHDZ53 | Sa10G479030 |
| Gmhdz79 | Glyma18g15970 | SaHDZ54 | Sa10G506180 |
| Gmhdz80 | Glyma18g16390 | SaHDZ55 | Sa11G523130 |
| Gmhdz81 | Glyma18g45970 | SaHDZ56 | Sa11G529010 |
| Gmhdz82 | Glyma18g48880 | SaHDZ57 | Sa11G552010 |
| Gmhdz83 | Glyma18g49290 | SaHDZ58 | Sa11G554720 |
| Gmhdz84 | Glyma19g01300 | SaHDZ59 | Sa11G558380 |
| Gmhdz85 | Glyma19g02490 | SaHDZ60 | Sa11G558800 |
| Gmhdz86 | Glyma19g33100 | SaHDZ61 | Sa12G565150 |
| Gmhdz87 | Glyma19g37380 | SaHDZ62 | Sa12G571340 |
| Gmhdz88 | Glyma20g01770 | SaHDZ63 | Sa12G595590 |
| HDG2 | AT1G05230 | SaHDZ64 | Sa12G598650 |
| HDG12 | AT1G17920 | SaHDZ65 | Sa12G602590 |
| ATHB23 | AT1G26960 | SaHDZ66 | Sa12G602800 |
| ATHB54 | AT1G27045 | SaHDZ67 | Sa13G629800 |
| ATHB9 | AT1G30490 | SaHDZ68 | Sa13G635330 |
| HDG10 | AT1G34650 | SaHDZ69 | Sa14G674730 |
| ATHB15 | AT1G52150 | SaHDZ70 | Sa14G680550 |
| ATHB13 | AT1G69780 | SaHDZ71 | Sa15G695940 |
| ATHB18 | AT1G70920 | SaHDZ72 | Sa15G697670 |
| HDG11 | AT1G73360 | SaHDZ73 | Sa15G710450 |
| ATHB10 | AT1G79840 | SaHDZ74 | Sa15G714720 |
| ATHB17 | AT2G01430 | SaHDZ75 | Sa15G737000 |
| ATHB21 | AT2G18550 | SaHDZ76 | Sa15G739230 |
| ATHB6 | AT2G22430 | SaHDZ77 | Sa16G747010 |
| HAT9 | AT2G22800 | SaHDZ78 | Sa16G748980 |
| HDG3 | AT2G32370 | SaHDZ79 | Sa16G763890 |
| ATHB14 | AT2G34710 | SaHDZ80 | Sa16G768280 |
| ATHB22 | AT2G36610 | SaHDZ81 | Sa16G792750 |
| ATHB4 | AT2G44910 | SaHDZ82 | Sa16G794550 |
| ATHB7 | AT2G46680 | SaHDZ83 | Sa17G805790 |
| ATHB20 | AT3G01220 | SaHDZ84 | Sa17G816310 |
| ATHB1 | AT3G01470 | SaHDZ85 | Sa18G840120 |
| HDG8 | AT3G03260 | SaHDZ86 | Sa18G850000 |

**Table S2** Primers used for detection and real-time PCR analyses

| actin-F | GTCCTTTCAGGAGGTACAACC |
| --- | --- |
| actin-R | CCACATCTGCTGGAAGGTGC |
| SaHDZ8-F | CACTTCCTCGCCGAACA |
| SaHDZ8-R | GCGAGCACGACGCCC |
| SaHDZ22-F | TGAAGTTGCAGTTGGCAAG |
| SaHDZ22-R | AAGTTGCTTTGACTTCCATCTA |
| SaHDZ27-F | GGTCTCGGCCTTCACGAT |
| SaHDZ27-R | GATTGATCCTCCTCCTTTTTC |
| SaHDZ30-F | GGGGGCATCATTCAGAGAAG |
| SaHDZ30-R | TTCAGGGTCAAGCTTGTTTT |
| SaHDZ58-F | AGAGTATAGAGCAGTGCAAGAT |
| SaHDZ58-R | CCCTACCCGTTAGGCCTAG |
| SaHDZ70-F | TGCAAGAAAGTGGCAGGAG |
| SaHDZ70-R | GGCTTCCGCAAGAGC |
| SaHDZ84-F | TTAATCGGAGAGTGCCCC |
| SaHDZ84-R | TCTTTTCTCTGCTTCTCCCT |
| SaHDZ71-F | TACTGAGGAGGAGGAGGAG |
| SaHDZ71-R | AGCCATCGGAAACATCCT |
| SaTPR-F | CCCACTTCCCTCACTCT |
| SaTPR-R | CCCTCCTTGAACATCCTCAAA |
| SaAKI-F | GACCAAACAACTTGAAAGGAAT |
| SaAKI-R | GCTTCTCATTCTCCTTCACAAT |

**Table S3** *SaHDZ22* sequence truncation statistics.

| No | Segment start and stop | Sequence length | Amino acid length |
| --- | --- | --- | --- |
| 1 | 1—738 | 738 bp | 245AA |
| 2 | 1—117 | 117 bp | 39AA |
| 3 | 1—267 | 267 bp | 89AA |
| 4 | 118—267 | 150 bp | 50AA |
| 5 | 1—399 | 399 bp | 133AA |
| 6 | 118—399 | 282 bp | 93AA |
| 7 | 268—399 | 132 bp | 43AA |
| 8 | 118—738 | 621 bp | 206AA |
| 9 | 268—738 | 471 bp | 156AA |
| 10 | 340—738 | 399 bp | 133AA |

**Table S4** Annotation of predictive interacting proteins.

| queryIndex | node1 | node2 | node1_string_id | node2_string_id | combined_score |
| --- | --- | --- | --- | --- | --- |
| 1 | ABF3 | CIPK11 | 3702.Q9M7Q3 | 3702.O22932 | 0.194 |
| 2 | ABF3 | GBF1 | 3702.Q9M7Q3 | 3702.P42774 | 0.407 |
| 3 | ABF3 | HAT22 | 3702.Q9M7Q3 | 3702.P46604 | 0.163 |
| 4 | ABF3 | ATHB-5 | 3702.Q9M7Q3 | 3702.P46667 | 0.282 |
| 5 | ABF3 | ATHB-6 | 3702.Q9M7Q3 | 3702.P46668 | 0.552 |
| 6 | ABF3 | ATHB-7 | 3702.Q9M7Q3 | 3702.P46897 | 0.768 |
| 7 | ABF3 | ABI1 | 3702.Q9M7Q3 | 3702.P49597 | 0.848 |
| 8 | ABF3 | LTI65 | 3702.Q9M7Q3 | 3702.Q04980 | 0.888 |
| 9 | ABF3 | MPK3 | 3702.Q9M7Q3 | 3702.Q39023 | 0.268 |
| 10 | ABF3 | NAC072 | 3702.Q9M7Q3 | 3702.Q93VY3 | 0.803 |
| 11 | ABF3 | SAG113 | 3702.Q9M7Q3 | 3702.Q9FIF5 | 0.713 |
| 12 | ABF3 | HDG11 | 3702.Q9M7Q3 | 3702.Q9FX31 | 0.226 |
| 13 | ABF3 | ATHB-12 | 3702.Q9M7Q3 | 3702.Q9M276 | 0.659 |
| 14 | ABI1 | CIPK11 | 3702.P49597 | 3702.O22932 | 0.656 |
| 15 | ABI1 | HAT22 | 3702.P49597 | 3702.P46604 | 0.176 |
| 16 | ABI1 | ATHB-5 | 3702.P49597 | 3702.P46667 | 0.285 |
| 17 | ABI1 | ATHB-6 | 3702.P49597 | 3702.P46668 | 0.93 |
| 18 | ABI1 | ATHB-7 | 3702.P49597 | 3702.P46897 | 0.572 |
| 19 | ABI1 | SPY | 3702.P49597 | 3702.Q96301 | 0.182 |
| 20 | ABI1 | PIN3 | 3702.P49597 | 3702.Q9S7Z8 | 0.2 |
| 21 | ABI1 | SAG113 | 3702.P49597 | 3702.Q9FIF5 | 0.467 |
| 22 | ABI1 | ATHB-12 | 3702.P49597 | 3702.Q9M276 | 0.579 |
| 23 | ABI1 | NAC072 | 3702.P49597 | 3702.Q93VY3 | 0.797 |
| 24 | ABI1 | MPK3 | 3702.P49597 | 3702.Q39023 | 0.823 |
| 25 | ABI1 | LTI65 | 3702.P49597 | 3702.Q04980 | 0.895 |
| 26 | AHL12 | HDG11 | 3702.Q8LPN5 | 3702.Q9FX31 | 0.455 |
| 27 | AHL12 | ATHB-12 | 3702.Q8LPN5 | 3702.Q9M276 | 0.57 |
| 28 | AIL6 | ATHB-14 | 3702.Q52QU2 | 3702.O04291 | 0.216 |
| 29 | AIL6 | GATA9 | 3702.Q52QU2 | 3702.O82632 | 0.548 |
| 30 | AIL6 | HAT14 | 3702.Q52QU2 | 3702.P46665 | 0.26 |
| 31 | AIL6 | MPK3 | 3702.Q52QU2 | 3702.Q39023 | 0.192 |
| 32 | AIL6 | ATHB-8 | 3702.Q52QU2 | 3702.Q39123 | 0.156 |
| 33 | AIL6 | STP14 | 3702.Q52QU2 | 3702.Q8GW61 | 0.174 |
| 34 | AIL6 | PIN3 | 3702.Q52QU2 | 3702.Q9S7Z8 | 0.199 |
| 35 | AIL6 | SLD5 | 3702.Q52QU2 | 3702.Q6NQP5 | 0.214 |
| 36 | AIL6 | APG-2 | 3702.Q52QU2 | 3702.Q9LU14 | 0.246 |
| 37 | AIL6 | GUS3 | 3702.Q52QU2 | 3702.Q9FZP1 | 0.597 |
| 38 | AMSH1 | ATHB-5 | 3702.Q8VYB5 | 3702.P46667 | 0.284 |
| 39 | AMSH1 | ATHB-6 | 3702.Q8VYB5 | 3702.P46668 | 0.284 |
| 40 | AMSH1 | AMSH3 | 3702.Q8VYB5 | 3702.Q5PNU3 | 0.91 |
| 41 | AMSH1 | AMSH2 | 3702.Q8VYB5 | 3702.Q6NKP9 | 0.906 |
| 42 | AMSH1 | CDKB2-2 | 3702.Q8VYB5 | 3702.Q8LG64 | 0.21 |
| 43 | AMSH1 | ATHB-16 | 3702.Q8VYB5 | 3702.Q940J1 | 0.236 |
| 44 | AMSH2 | ATHB-5 | 3702.Q6NKP9 | 3702.P46667 | 0.242 |
| 45 | AMSH2 | ATHB-6 | 3702.Q6NKP9 | 3702.P46668 | 0.332 |
| 46 | AMSH2 | AMSH3 | 3702.Q6NKP9 | 3702.Q5PNU3 | 0.906 |
| 47 | AMSH2 | CDKB2-2 | 3702.Q6NKP9 | 3702.Q8LG64 | 0.21 |
| 48 | AMSH2 | ATHB-16 | 3702.Q6NKP9 | 3702.Q940J1 | 0.218 |
| 49 | AMSH3 | ATHB-5 | 3702.Q5PNU3 | 3702.P46667 | 0.288 |
| 50 | AMSH3 | ATHB-6 | 3702.Q5PNU3 | 3702.P46668 | 0.288 |
| 51 | AMSH3 | CDKB2-2 | 3702.Q5PNU3 | 3702.Q8LG64 | 0.21 |
| 52 | AMSH3 | ATHB-16 | 3702.Q5PNU3 | 3702.Q940J1 | 0.239 |
| 53 | ANL2 | F4J1U2_ARATH | 3702.Q0WV12 | 3702.F4J1U2 | 0.333 |
| 54 | ANL2 | F4J3A9_ARATH | 3702.Q0WV12 | 3702.F4J3A9 | 0.175 |
| 55 | ANL2 | ATHB-14 | 3702.Q0WV12 | 3702.O04291 | 0.206 |
| 56 | ANL2 | PRA1B4 | 3702.Q0WV12 | 3702.O80915 | 0.151 |
| 57 | ANL2 | GATA9 | 3702.Q0WV12 | 3702.O82632 | 0.628 |
| 58 | ANL2 | HAT3 | 3702.Q0WV12 | 3702.P46602 | 0.288 |
| 59 | ANL2 | HAT22 | 3702.Q0WV12 | 3702.P46604 | 0.477 |
| 60 | ANL2 | GL2 | 3702.Q0WV12 | 3702.P46607 | 0.16 |
| 61 | ANL2 | HAT14 | 3702.Q0WV12 | 3702.P46665 | 0.279 |
| 62 | ANL2 | ATHB-5 | 3702.Q0WV12 | 3702.P46667 | 0.433 |
| 63 | ANL2 | ATHB-6 | 3702.Q0WV12 | 3702.P46668 | 0.333 |
| 64 | ANL2 | ATHB-7 | 3702.Q0WV12 | 3702.P46897 | 0.543 |
| 65 | ANL2 | HAT4 | 3702.Q0WV12 | 3702.Q05466 | 0.542 |
| 66 | ANL2 | AT1G53040.1 | 3702.Q0WV12 | 3702.Q0WNW2 | 0.295 |
| 67 | ANL2 | PDF2 | 3702.Q0WV12 | 3702.Q93V99 | 0.165 |
| 68 | ANL2 | ATHB-13 | 3702.Q0WV12 | 3702.Q8LC03 | 0.165 |
| 69 | ANL2 | T14D3.150 | 3702.Q0WV12 | 3702.Q9M1T8 | 0.191 |
| 70 | ANL2 | CDKB2-2 | 3702.Q0WV12 | 3702.Q8LG64 | 0.197 |
| 71 | ANL2 | ATHB-16 | 3702.Q0WV12 | 3702.Q940J1 | 0.199 |
| 72 | ANL2 | SPY | 3702.Q0WV12 | 3702.Q96301 | 0.2 |
| 73 | ANL2 | ATML1 | 3702.Q0WV12 | 3702.Q8RWU4 | 0.203 |
| 74 | ANL2 | Q9LSC7_ARATH | 3702.Q0WV12 | 3702.Q9LSC7 | 0.237 |
| 75 | ANL2 | STP14 | 3702.Q0WV12 | 3702.Q8GW61 | 0.238 |
| 76 | ANL2 | SLD5 | 3702.Q0WV12 | 3702.Q6NQP5 | 0.278 |
| 77 | ANL2 | DRB2 | 3702.Q0WV12 | 3702.Q9SKN2 | 0.297 |
| 78 | ANL2 | AT5G42660.1 | 3702.Q0WV12 | 3702.Q93Z91 | 0.316 |
| 79 | ANL2 | APG-2 | 3702.Q0WV12 | 3702.Q9LU14 | 0.371 |
| 80 | ANL2 | ATHB-52 | 3702.Q0WV12 | 3702.Q9FN29 | 0.397 |
| 81 | ANL2 | GUS3 | 3702.Q0WV12 | 3702.Q9FZP1 | 0.4 |
| 82 | ANL2 | F9F8.20 | 3702.Q0WV12 | 3702.Q8W4N1 | 0.408 |
| 83 | ANL2 | PIN3 | 3702.Q0WV12 | 3702.Q9S7Z8 | 0.413 |
| 84 | ANL2 | ATHB-12 | 3702.Q0WV12 | 3702.Q9M276 | 0.437 |
| 85 | ANL2 | ATHB-15 | 3702.Q0WV12 | 3702.Q9ZU11 | 0.445 |
| 86 | ANL2 | ATHB-8 | 3702.Q0WV12 | 3702.Q39123 | 0.456 |
| 87 | ANL2 | Q8S8Q2_ARATH | 3702.Q0WV12 | 3702.Q8S8Q2 | 0.482 |
| 88 | APG-2 | PRA1B4 | 3702.Q9LU14 | 3702.O80915 | 0.307 |
| 89 | APG-2 | GATA9 | 3702.Q9LU14 | 3702.O82632 | 0.54 |
| 90 | APG-2 | HAT4 | 3702.Q9LU14 | 3702.Q05466 | 0.193 |
| 91 | APG-2 | SLD5 | 3702.Q9LU14 | 3702.Q6NQP5 | 0.483 |
| 92 | APG-2 | STP14 | 3702.Q9LU14 | 3702.Q8GW61 | 0.4 |
| 93 | APG-2 | CDKB2-2 | 3702.Q9LU14 | 3702.Q8LG64 | 0.324 |
| 94 | APG-2 | GUS3 | 3702.Q9LU14 | 3702.Q9FZP1 | 0.637 |
| 95 | AT1G53040.1 | ATHB-14 | 3702.Q0WNW2 | 3702.O04291 | 0.341 |
| 96 | AT1G53040.1 | HDG2 | 3702.Q0WNW2 | 3702.Q94C37 | 0.265 |
| 97 | AT1G53040.1 | ATML1 | 3702.Q0WNW2 | 3702.Q8RWU4 | 0.293 |
| 98 | AT1G53040.1 | PDF2 | 3702.Q0WNW2 | 3702.Q93V99 | 0.298 |
| 99 | AT1G53040.1 | Q8S8Q2_ARATH | 3702.Q0WNW2 | 3702.Q8S8Q2 | 0.326 |
| 100 | AT1G53040.1 | DRB2 | 3702.Q0WNW2 | 3702.Q9SKN2 | 0.37 |
| 101 | AT1G53040.1 | F9F8.20 | 3702.Q0WNW2 | 3702.Q8W4N1 | 0.388 |
| 102 | AT1G53040.1 | ATHB-8 | 3702.Q0WNW2 | 3702.Q39123 | 0.421 |
| 103 | AT1G53040.1 | ATHB-15 | 3702.Q0WNW2 | 3702.Q9ZU11 | 0.441 |
| 104 | AT5G42660.1 | F4J1U2_ARATH | 3702.Q93Z91 | 3702.F4J1U2 | 0.32 |
| 105 | AT5G42660.1 | ATHB-14 | 3702.Q93Z91 | 3702.O04291 | 0.444 |
| 106 | AT5G42660.1 | GL2 | 3702.Q93Z91 | 3702.P46607 | 0.26 |
| 107 | AT5G42660.1 | ATHB-8 | 3702.Q93Z91 | 3702.Q39123 | 0.467 |
| 108 | AT5G42660.1 | ATML1 | 3702.Q93Z91 | 3702.Q8RWU4 | 0.314 |
| 109 | AT5G42660.1 | Q8S8Q2_ARATH | 3702.Q93Z91 | 3702.Q8S8Q2 | 0.379 |
| 110 | AT5G42660.1 | F9F8.20 | 3702.Q93Z91 | 3702.Q8W4N1 | 0.448 |
| 111 | AT5G42660.1 | PDF2 | 3702.Q93Z91 | 3702.Q93V99 | 0.33 |
| 112 | AT5G42660.1 | Q9LSC7_ARATH | 3702.Q93Z91 | 3702.Q9LSC7 | 0.231 |
| 113 | AT5G42660.1 | HDG11 | 3702.Q93Z91 | 3702.Q9FX31 | 0.279 |
| 114 | AT5G42660.1 | HDG2 | 3702.Q93Z91 | 3702.Q94C37 | 0.3 |
| 115 | AT5G42660.1 | DRB2 | 3702.Q93Z91 | 3702.Q9SKN2 | 0.345 |
| 116 | AT5G42660.1 | ATHB-15 | 3702.Q93Z91 | 3702.Q9ZU11 | 0.483 |
| 117 | ATH1 | HAT3 | 3702.P48731 | 3702.P46602 | 0.41 |
| 118 | ATH1 | HAT22 | 3702.P48731 | 3702.P46604 | 0.192 |
| 119 | ATH1 | HAT14 | 3702.P48731 | 3702.P46665 | 0.567 |
| 120 | ATH1 | ATHB-5 | 3702.P48731 | 3702.P46667 | 0.187 |
| 121 | ATH1 | ATHB-6 | 3702.P48731 | 3702.P46668 | 0.187 |
| 122 | ATH1 | ATHB-7 | 3702.P48731 | 3702.P46897 | 0.187 |
| 123 | ATH1 | HDG2 | 3702.P48731 | 3702.Q94C37 | 0.153 |
| 124 | ATH1 | ATML1 | 3702.P48731 | 3702.Q8RWU4 | 0.154 |
| 125 | ATH1 | PDF2 | 3702.P48731 | 3702.Q93V99 | 0.163 |
| 126 | ATH1 | ATHB-52 | 3702.P48731 | 3702.Q9FN29 | 0.187 |
| 127 | ATH1 | ATHB-12 | 3702.P48731 | 3702.Q9M276 | 0.187 |
| 128 | ATH1 | ATHB-16 | 3702.P48731 | 3702.Q940J1 | 0.187 |
| 129 | ATH1 | ATHB-13 | 3702.P48731 | 3702.Q8LC03 | 0.19 |
| 130 | ATH1 | HAT4 | 3702.P48731 | 3702.Q05466 | 0.411 |
| 131 | ATHB-12 | ATHB-14 | 3702.Q9M276 | 3702.O04291 | 0.321 |
| 132 | ATHB-12 | CIPK11 | 3702.Q9M276 | 3702.O22932 | 0.385 |
| 133 | ATHB-12 | PRA1B4 | 3702.Q9M276 | 3702.O80915 | 0.35 |
| 134 | ATHB-12 | HAT3 | 3702.Q9M276 | 3702.P46602 | 0.282 |
| 135 | ATHB-12 | HAT22 | 3702.Q9M276 | 3702.P46604 | 0.247 |
| 136 | ATHB-12 | GL2 | 3702.Q9M276 | 3702.P46607 | 0.156 |
| 137 | ATHB-12 | ATHB-5 | 3702.Q9M276 | 3702.P46667 | 0.469 |
| 138 | ATHB-12 | ATHB-6 | 3702.Q9M276 | 3702.P46668 | 0.249 |
| 139 | ATHB-12 | ATHB-7 | 3702.Q9M276 | 3702.P46897 | 0.696 |
| 140 | ATHB-12 | LTI65 | 3702.Q9M276 | 3702.Q04980 | 0.545 |
| 141 | ATHB-12 | HAT4 | 3702.Q9M276 | 3702.Q05466 | 0.349 |
| 142 | ATHB-12 | F14F18.170 | 3702.Q9M276 | 3702.Q0WVJ0 | 0.473 |
| 143 | ATHB-12 | ATHB-8 | 3702.Q9M276 | 3702.Q39123 | 0.718 |
| 144 | ATHB-12 | CSC1 | 3702.Q9M276 | 3702.Q5XEZ5 | 0.152 |
| 145 | ATHB-12 | ATHB-13 | 3702.Q9M276 | 3702.Q8LC03 | 0.221 |
| 146 | ATHB-12 | ATML1 | 3702.Q9M276 | 3702.Q8RWU4 | 0.545 |
| 147 | ATHB-12 | NAC072 | 3702.Q9M276 | 3702.Q93VY3 | 0.802 |
| 148 | ATHB-12 | ATHB-16 | 3702.Q9M276 | 3702.Q940J1 | 0.189 |
| 149 | ATHB-12 | SAG113 | 3702.Q9M276 | 3702.Q9FIF5 | 0.619 |
| 150 | ATHB-12 | ATHB-52 | 3702.Q9M276 | 3702.Q9FN29 | 0.191 |
| 151 | ATHB-12 | HDG11 | 3702.Q9M276 | 3702.Q9FX31 | 0.301 |
| 152 | ATHB-12 | BGAL13 | 3702.Q9M276 | 3702.Q9SCU9 | 0.223 |
| 153 | ATHB-12 | ATHB-15 | 3702.Q9M276 | 3702.Q9ZU11 | 0.431 |
| 154 | ATHB-12 | RKP | 3702.Q9M276 | 3702.Q9SIZ8 | 0.635 |
| 155 | ATHB-13 | ATHB-14 | 3702.Q8LC03 | 3702.O04291 | 0.151 |
| 156 | ATHB-13 | HAT3 | 3702.Q8LC03 | 3702.P46602 | 0.173 |
| 157 | ATHB-13 | GL2 | 3702.Q8LC03 | 3702.P46607 | 0.157 |
| 158 | ATHB-13 | ATHB-5 | 3702.Q8LC03 | 3702.P46667 | 0.277 |
| 159 | ATHB-13 | ATHB-6 | 3702.Q8LC03 | 3702.P46668 | 0.276 |
| 160 | ATHB-13 | ATHB-7 | 3702.Q8LC03 | 3702.P46897 | 0.294 |
| 161 | ATHB-13 | ATHB-8 | 3702.Q8LC03 | 3702.Q39123 | 0.252 |
| 162 | ATHB-13 | ATHB-52 | 3702.Q8LC03 | 3702.Q9FN29 | 0.155 |
| 163 | ATHB-13 | ATML1 | 3702.Q8LC03 | 3702.Q8RWU4 | 0.197 |
| 164 | ATHB-13 | ATHB-16 | 3702.Q8LC03 | 3702.Q940J1 | 0.255 |
| 165 | ATHB-13 | HDG11 | 3702.Q8LC03 | 3702.Q9FX31 | 0.262 |
| 166 | ATHB-13 | PDF2 | 3702.Q8LC03 | 3702.Q93V99 | 0.267 |
| 167 | ATHB-13 | ATHB-15 | 3702.Q8LC03 | 3702.Q9ZU11 | 0.716 |
| 168 | ATHB-14 | F4J1U2_ARATH | 3702.O04291 | 3702.F4J1U2 | 0.329 |
| 169 | ATHB-14 | PIN3 | 3702.O04291 | 3702.Q9S7Z8 | 0.171 |
| 170 | ATHB-14 | PDF2 | 3702.O04291 | 3702.Q93V99 | 0.244 |
| 171 | ATHB-14 | ATHB-8 | 3702.O04291 | 3702.Q39123 | 0.245 |
| 172 | ATHB-14 | HDG2 | 3702.O04291 | 3702.Q94C37 | 0.251 |
| 173 | ATHB-14 | HDG11 | 3702.O04291 | 3702.Q9FX31 | 0.28 |
| 174 | ATHB-14 | ATHB-7 | 3702.O04291 | 3702.P46897 | 0.305 |
| 175 | ATHB-14 | ATHB-5 | 3702.O04291 | 3702.P46667 | 0.321 |
| 176 | ATHB-14 | ATHB-15 | 3702.O04291 | 3702.Q9ZU11 | 0.334 |
| 177 | ATHB-14 | ATML1 | 3702.O04291 | 3702.Q8RWU4 | 0.351 |
| 178 | ATHB-14 | F9F8.20 | 3702.O04291 | 3702.Q8W4N1 | 0.396 |
| 179 | ATHB-14 | DRB2 | 3702.O04291 | 3702.Q9SKN2 | 0.408 |
| 180 | ATHB-14 | Q8S8Q2_ARATH | 3702.O04291 | 3702.Q8S8Q2 | 0.445 |
| 181 | ATHB-14 | DCP2 | 3702.O04291 | 3702.Q8GW31 | 0.451 |
| 182 | ATHB-14 | HAT4 | 3702.O04291 | 3702.Q05466 | 0.542 |
| 183 | ATHB-14 | HAT3 | 3702.O04291 | 3702.P46602 | 0.666 |
| 184 | ATHB-15 | F4J1U2_ARATH | 3702.Q9ZU11 | 3702.F4J1U2 | 0.321 |
| 185 | ATHB-15 | HAT3 | 3702.Q9ZU11 | 3702.P46602 | 0.549 |
| 186 | ATHB-15 | HAT22 | 3702.Q9ZU11 | 3702.P46604 | 0.197 |
| 187 | ATHB-15 | GL2 | 3702.Q9ZU11 | 3702.P46607 | 0.31 |
| 188 | ATHB-15 | ATHB-5 | 3702.Q9ZU11 | 3702.P46667 | 0.571 |
| 189 | ATHB-15 | ATHB-6 | 3702.Q9ZU11 | 3702.P46668 | 0.228 |
| 190 | ATHB-15 | ATHB-7 | 3702.Q9ZU11 | 3702.P46897 | 0.319 |
| 191 | ATHB-15 | HAT4 | 3702.Q9ZU11 | 3702.Q05466 | 0.656 |
| 192 | ATHB-15 | ATHB-8 | 3702.Q9ZU11 | 3702.Q39123 | 0.328 |
| 193 | ATHB-15 | DCP2 | 3702.Q9ZU11 | 3702.Q8GW31 | 0.451 |
| 194 | ATHB-15 | ATML1 | 3702.Q9ZU11 | 3702.Q8RWU4 | 0.428 |
| 195 | ATHB-15 | Q8S8Q2_ARATH | 3702.Q9ZU11 | 3702.Q8S8Q2 | 0.429 |
| 196 | ATHB-15 | F9F8.20 | 3702.Q9ZU11 | 3702.Q8W4N1 | 0.433 |
| 197 | ATHB-15 | PDF2 | 3702.Q9ZU11 | 3702.Q93V99 | 0.369 |
| 198 | ATHB-15 | ATHB-16 | 3702.Q9ZU11 | 3702.Q940J1 | 0.229 |
| 199 | ATHB-15 | HDG2 | 3702.Q9ZU11 | 3702.Q94C37 | 0.25 |
| 200 | ATHB-15 | ATHB-52 | 3702.Q9ZU11 | 3702.Q9FN29 | 0.309 |
| 201 | ATHB-15 | HDG11 | 3702.Q9ZU11 | 3702.Q9FX31 | 0.343 |
| 202 | ATHB-15 | Q9LSC7_ARATH | 3702.Q9ZU11 | 3702.Q9LSC7 | 0.287 |
| 203 | ATHB-15 | PIN3 | 3702.Q9ZU11 | 3702.Q9S7Z8 | 0.304 |
| 204 | ATHB-15 | DRB2 | 3702.Q9ZU11 | 3702.Q9SKN2 | 0.375 |
| 205 | ATHB-16 | HAT3 | 3702.Q940J1 | 3702.P46602 | 0.28 |
| 206 | ATHB-16 | HAT22 | 3702.Q940J1 | 3702.P46604 | 0.213 |
| 207 | ATHB-16 | HAT14 | 3702.Q940J1 | 3702.P46665 | 0.241 |
| 208 | ATHB-16 | ATHB-5 | 3702.Q940J1 | 3702.P46667 | 0.407 |
| 209 | ATHB-16 | ATHB-7 | 3702.Q940J1 | 3702.P46897 | 0.209 |
| 210 | ATHB-16 | HAT4 | 3702.Q940J1 | 3702.Q05466 | 0.293 |
| 211 | ATHB-16 | ATHB-8 | 3702.Q940J1 | 3702.Q39123 | 0.211 |
| 212 | ATHB-16 | ATML1 | 3702.Q940J1 | 3702.Q8RWU4 | 0.33 |
| 213 | ATHB-16 | ATHB-52 | 3702.Q940J1 | 3702.Q9FN29 | 0.161 |
| 214 | ATHB-16 | HDG11 | 3702.Q940J1 | 3702.Q9FX31 | 0.244 |
| 215 | ATHB-5 | HAT3 | 3702.P46667 | 3702.P46602 | 0.363 |
| 216 | ATHB-5 | HAT22 | 3702.P46667 | 3702.P46604 | 0.222 |
| 217 | ATHB-5 | GL2 | 3702.P46667 | 3702.P46607 | 0.175 |
| 218 | ATHB-5 | HAT14 | 3702.P46667 | 3702.P46665 | 0.203 |
| 219 | ATHB-5 | ATHB-52 | 3702.P46667 | 3702.Q9FN29 | 0.172 |
| 220 | ATHB-5 | NAC072 | 3702.P46667 | 3702.Q93VY3 | 0.195 |
| 221 | ATHB-5 | PDF2 | 3702.P46667 | 3702.Q93V99 | 0.285 |
| 222 | ATHB-5 | HDG11 | 3702.P46667 | 3702.Q9FX31 | 0.355 |
| 223 | ATHB-5 | HAT4 | 3702.P46667 | 3702.Q05466 | 0.378 |
| 224 | ATHB-5 | ATHB-8 | 3702.P46667 | 3702.Q39123 | 0.434 |
| 225 | ATHB-5 | ATHB-6 | 3702.P46667 | 3702.P46668 | 0.474 |
| 226 | ATHB-5 | ATHB-7 | 3702.P46667 | 3702.P46897 | 0.483 |
| 227 | ATHB-5 | ATML1 | 3702.P46667 | 3702.Q8RWU4 | 0.742 |
| 228 | ATHB-52 | ATHB-6 | 3702.Q9FN29 | 3702.P46668 | 0.183 |
| 229 | ATHB-52 | ATHB-7 | 3702.Q9FN29 | 3702.P46897 | 0.201 |
| 230 | ATHB-52 | HAT4 | 3702.Q9FN29 | 3702.Q05466 | 0.19 |
| 231 | ATHB-52 | ATHB-8 | 3702.Q9FN29 | 3702.Q39123 | 0.33 |
| 232 | ATHB-52 | ATML1 | 3702.Q9FN29 | 3702.Q8RWU4 | 0.601 |
| 233 | ATHB-52 | PDF2 | 3702.Q9FN29 | 3702.Q93V99 | 0.207 |
| 234 | ATHB-52 | NAC072 | 3702.Q9FN29 | 3702.Q93VY3 | 0.163 |
| 235 | ATHB-52 | WAG1 | 3702.Q9FN29 | 3702.Q9C8M5 | 0.785 |
| 236 | ATHB-52 | PIN3 | 3702.Q9FN29 | 3702.Q9S7Z8 | 0.165 |
| 237 | ATHB-52 | HDG11 | 3702.Q9FN29 | 3702.Q9FX31 | 0.552 |
| 238 | ATHB-6 | CIPK11 | 3702.P46668 | 3702.O22932 | 0.426 |
| 239 | ATHB-6 | HAT3 | 3702.P46668 | 3702.P46602 | 0.262 |
| 240 | ATHB-6 | HAT22 | 3702.P46668 | 3702.P46604 | 0.309 |
| 241 | ATHB-6 | GL2 | 3702.P46668 | 3702.P46607 | 0.197 |
| 242 | ATHB-6 | HAT14 | 3702.P46668 | 3702.P46665 | 0.228 |
| 243 | ATHB-6 | PDF2 | 3702.P46668 | 3702.Q93V99 | 0.16 |
| 244 | ATHB-6 | NAC072 | 3702.P46668 | 3702.Q93VY3 | 0.254 |
| 245 | ATHB-6 | HAT4 | 3702.P46668 | 3702.Q05466 | 0.262 |
| 246 | ATHB-6 | ATHB-8 | 3702.P46668 | 3702.Q39123 | 0.263 |
| 247 | ATHB-6 | ATHB-7 | 3702.P46668 | 3702.P46897 | 0.287 |
| 248 | ATHB-6 | HDG11 | 3702.P46668 | 3702.Q9FX31 | 0.35 |
| 249 | ATHB-6 | SAG113 | 3702.P46668 | 3702.Q9FIF5 | 0.38 |
| 250 | ATHB-6 | ATML1 | 3702.P46668 | 3702.Q8RWU4 | 0.543 |
| 251 | ATHB-7 | CIPK11 | 3702.P46897 | 3702.O22932 | 0.165 |
| 252 | ATHB-7 | HAT3 | 3702.P46897 | 3702.P46602 | 0.264 |
| 253 | ATHB-7 | HAT22 | 3702.P46897 | 3702.P46604 | 0.279 |
| 254 | ATHB-7 | GL2 | 3702.P46897 | 3702.P46607 | 0.156 |
| 255 | ATHB-7 | HAT4 | 3702.P46897 | 3702.Q05466 | 0.305 |
| 256 | ATHB-7 | ATML1 | 3702.P46897 | 3702.Q8RWU4 | 0.433 |
| 257 | ATHB-7 | SAG113 | 3702.P46897 | 3702.Q9FIF5 | 0.524 |
| 258 | ATHB-7 | LTI65 | 3702.P46897 | 3702.Q04980 | 0.561 |
| 259 | ATHB-7 | ATHB-8 | 3702.P46897 | 3702.Q39123 | 0.599 |
| 260 | ATHB-7 | HDG11 | 3702.P46897 | 3702.Q9FX31 | 0.632 |
| 261 | ATHB-7 | RKP | 3702.P46897 | 3702.Q9SIZ8 | 0.677 |
| 262 | ATHB-7 | NAC072 | 3702.P46897 | 3702.Q93VY3 | 0.783 |
| 263 | ATHB-8 | F4J1U2_ARATH | 3702.Q39123 | 3702.F4J1U2 | 0.314 |
| 264 | ATHB-8 | HAT3 | 3702.Q39123 | 3702.P46602 | 0.342 |
| 265 | ATHB-8 | HAT22 | 3702.Q39123 | 3702.P46604 | 0.319 |
| 266 | ATHB-8 | GL2 | 3702.Q39123 | 3702.P46607 | 0.405 |
| 267 | ATHB-8 | HAT4 | 3702.Q39123 | 3702.Q05466 | 0.776 |
| 268 | ATHB-8 | Q9LSC7_ARATH | 3702.Q39123 | 3702.Q9LSC7 | 0.217 |
| 269 | ATHB-8 | HDG2 | 3702.Q39123 | 3702.Q94C37 | 0.234 |
| 270 | ATHB-8 | DRB2 | 3702.Q39123 | 3702.Q9SKN2 | 0.288 |
| 271 | ATHB-8 | HDG11 | 3702.Q39123 | 3702.Q9FX31 | 0.361 |
| 272 | ATHB-8 | Q8S8Q2_ARATH | 3702.Q39123 | 3702.Q8S8Q2 | 0.389 |
| 273 | ATHB-8 | F9F8.20 | 3702.Q39123 | 3702.Q8W4N1 | 0.398 |
| 274 | ATHB-8 | PDF2 | 3702.Q39123 | 3702.Q93V99 | 0.424 |
| 275 | ATHB-8 | PIN3 | 3702.Q39123 | 3702.Q9S7Z8 | 0.468 |
| 276 | ATHB-8 | ATML1 | 3702.Q39123 | 3702.Q8RWU4 | 0.473 |
| 277 | ATML1 | F4J1U2_ARATH | 3702.Q8RWU4 | 3702.F4J1U2 | 0.39 |
| 278 | ATML1 | F4J3A9_ARATH | 3702.Q8RWU4 | 3702.F4J3A9 | 0.302 |
| 279 | ATML1 | GATA2 | 3702.Q8RWU4 | 3702.O49741 | 0.168 |
| 280 | ATML1 | HAT3 | 3702.Q8RWU4 | 3702.P46602 | 0.599 |
| 281 | ATML1 | HAT22 | 3702.Q8RWU4 | 3702.P46604 | 0.205 |
| 282 | ATML1 | GL2 | 3702.Q8RWU4 | 3702.P46607 | 0.157 |
| 283 | ATML1 | HAT14 | 3702.Q8RWU4 | 3702.P46665 | 0.323 |
| 284 | ATML1 | HAT4 | 3702.Q8RWU4 | 3702.Q05466 | 0.66 |
| 285 | ATML1 | MPK3 | 3702.Q8RWU4 | 3702.Q39023 | 0.285 |
| 286 | ATML1 | HDG11 | 3702.Q8RWU4 | 3702.Q9FX31 | 0.156 |
| 287 | ATML1 | DRB2 | 3702.Q8RWU4 | 3702.Q9SKN2 | 0.257 |
| 288 | ATML1 | PIN3 | 3702.Q8RWU4 | 3702.Q9S7Z8 | 0.418 |
| 289 | ATML1 | F9F8.20 | 3702.Q8RWU4 | 3702.Q8W4N1 | 0.441 |
| 290 | ATML1 | Q8S8Q2_ARATH | 3702.Q8RWU4 | 3702.Q8S8Q2 | 0.514 |
| 291 | ATML1 | PDF2 | 3702.Q8RWU4 | 3702.Q93V99 | 0.707 |
| 292 | BGAL13 | PRA1B4 | 3702.Q9SCU9 | 3702.O80915 | 0.719 |
| 293 | BGAL13 | F14F18.170 | 3702.Q9SCU9 | 3702.Q0WVJ0 | 0.311 |
| 294 | BGAL13 | CSC1 | 3702.Q9SCU9 | 3702.Q5XEZ5 | 0.328 |
| 295 | BGAL13 | GUS3 | 3702.Q9SCU9 | 3702.Q9FZP1 | 0.161 |
| 296 | CDKB2-2 | CIPK11 | 3702.Q8LG64 | 3702.O22932 | 0.166 |
| 297 | CDKB2-2 | GATA9 | 3702.Q8LG64 | 3702.O82632 | 0.171 |
| 298 | CDKB2-2 | GL2 | 3702.Q8LG64 | 3702.P46607 | 0.275 |
| 299 | CDKB2-2 | MPK3 | 3702.Q8LG64 | 3702.Q39023 | 0.384 |
| 300 | CDKB2-2 | SLD5 | 3702.Q8LG64 | 3702.Q6NQP5 | 0.348 |
| 301 | CDKB2-2 | GUS3 | 3702.Q8LG64 | 3702.Q9FZP1 | 0.198 |
| 302 | CDKB2-2 | WAG1 | 3702.Q8LG64 | 3702.Q9C8M5 | 0.31 |
| 303 | CDKB2-2 | RKP | 3702.Q8LG64 | 3702.Q9SIZ8 | 0.595 |
| 304 | CIPK11 | F23N14.40 | 3702.O22932 | 3702.Q9M2C0 | 0.161 |
| 305 | CIPK11 | F14F18.170 | 3702.O22932 | 3702.Q0WVJ0 | 0.195 |
| 306 | CIPK11 | NAC072 | 3702.O22932 | 3702.Q93VY3 | 0.212 |
| 307 | CIPK11 | WAG1 | 3702.O22932 | 3702.Q9C8M5 | 0.26 |
| 308 | CIPK11 | MPK3 | 3702.O22932 | 3702.Q39023 | 0.274 |
| 309 | CIPK11 | SAG113 | 3702.O22932 | 3702.Q9FIF5 | 0.275 |
| 310 | CIPK11 | LTI65 | 3702.O22932 | 3702.Q04980 | 0.305 |
| 311 | CSC1 | PRA1B4 | 3702.Q5XEZ5 | 3702.O80915 | 0.598 |
| 312 | CSC1 | NRAMP6 | 3702.Q5XEZ5 | 3702.Q9S9N8 | 0.164 |
| 313 | CSC1 | NRAMP3 | 3702.Q5XEZ5 | 3702.Q9SNV9 | 0.164 |
| 314 | CSC1 | NRAMP2 | 3702.Q5XEZ5 | 3702.Q9C6B2 | 0.164 |
| 315 | DCP2 | RKP | 3702.Q8GW31 | 3702.Q9SIZ8 | 0.167 |
| 316 | DGK3 | MPK3 | 3702.Q8VZG1 | 3702.Q39023 | 0.152 |
| 317 | DGK3 | DGK5 | 3702.Q8VZG1 | 3702.Q9C5E5 | 0.288 |
| 318 | DGK5 | DGK7 | 3702.Q9C5E5 | 3702.F4JQ95 | 0.253 |
| 319 | DGK5 | F14F18.170 | 3702.Q9C5E5 | 3702.Q0WVJ0 | 0.745 |
| 320 | DGK5 | MPK3 | 3702.Q9C5E5 | 3702.Q39023 | 0.173 |
| 321 | DRB2 | F4J1U2_ARATH | 3702.Q9SKN2 | 3702.F4J1U2 | 0.284 |
| 322 | DRB2 | Q8S8Q2_ARATH | 3702.Q9SKN2 | 3702.Q8S8Q2 | 0.398 |
| 323 | DRB2 | F9F8.20 | 3702.Q9SKN2 | 3702.Q8W4N1 | 0.363 |
| 324 | DRB2 | PDF2 | 3702.Q9SKN2 | 3702.Q93V99 | 0.252 |
| 325 | DRB2 | HDG2 | 3702.Q9SKN2 | 3702.Q94C37 | 0.248 |
| 326 | DRB2 | Q9LSC7_ARATH | 3702.Q9SKN2 | 3702.Q9LSC7 | 0.205 |
| 327 | DRB2 | T14D3.150 | 3702.Q9SKN2 | 3702.Q9M1T8 | 0.224 |
| 328 | DRB2 | NRAMP6 | 3702.Q9SKN2 | 3702.Q9S9N8 | 0.293 |
| 329 | F4J1U2_ARATH | HDG11 | 3702.F4J1U2 | 3702.Q9FX31 | 0.201 |
| 330 | F4J1U2_ARATH | GL2 | 3702.F4J1U2 | 3702.P46607 | 0.212 |
| 331 | F4J1U2_ARATH | PIN3 | 3702.F4J1U2 | 3702.Q9S7Z8 | 0.247 |
| 332 | F4J1U2_ARATH | HDG2 | 3702.F4J1U2 | 3702.Q94C37 | 0.381 |
| 333 | F4J1U2_ARATH | F9F8.20 | 3702.F4J1U2 | 3702.Q8W4N1 | 0.398 |
| 334 | F4J1U2_ARATH | PDF2 | 3702.F4J1U2 | 3702.Q93V99 | 0.405 |
| 335 | F4J1U2_ARATH | Q8S8Q2_ARATH | 3702.F4J1U2 | 3702.Q8S8Q2 | 0.481 |
| 336 | F4J3A9_ARATH | HDG11 | 3702.F4J3A9 | 3702.Q9FX31 | 0.175 |
| 337 | F4J3A9_ARATH | HDG2 | 3702.F4J3A9 | 3702.Q94C37 | 0.302 |
| 338 | F4J3A9_ARATH | PDF2 | 3702.F4J3A9 | 3702.Q93V99 | 0.302 |
| 339 | F4J3A9_ARATH | GL2 | 3702.F4J3A9 | 3702.P46607 | 0.302 |
| 340 | F4J3A9_ARATH | MPK3 | 3702.F4J3A9 | 3702.Q39023 | 0.496 |
| 341 | F9F8.20 | GL2 | 3702.Q8W4N1 | 3702.P46607 | 0.21 |
| 342 | F9F8.20 | Q8S8Q2_ARATH | 3702.Q8W4N1 | 3702.Q8S8Q2 | 0.526 |
| 343 | F9F8.20 | Q9LSC7_ARATH | 3702.Q8W4N1 | 3702.Q9LSC7 | 0.248 |
| 344 | F9F8.20 | PIN3 | 3702.Q8W4N1 | 3702.Q9S7Z8 | 0.274 |
| 345 | F9F8.20 | HDG11 | 3702.Q8W4N1 | 3702.Q9FX31 | 0.409 |
| 346 | F9F8.20 | HDG2 | 3702.Q8W4N1 | 3702.Q94C37 | 0.433 |
| 347 | F9F8.20 | PDF2 | 3702.Q8W4N1 | 3702.Q93V99 | 0.452 |
| 348 | GATA2 | PDF2 | 3702.O49741 | 3702.Q93V99 | 0.168 |
| 349 | GATA2 | HDG2 | 3702.O49741 | 3702.Q94C37 | 0.168 |
| 350 | GATA2 | GL2 | 3702.O49741 | 3702.P46607 | 0.188 |
| 351 | GATA2 | GATA9 | 3702.O49741 | 3702.O82632 | 0.191 |
| 352 | GATA9 | PRA1B4 | 3702.O82632 | 3702.O80915 | 0.268 |
| 353 | GATA9 | GL2 | 3702.O82632 | 3702.P46607 | 0.193 |
| 354 | GATA9 | STP14 | 3702.O82632 | 3702.Q8GW61 | 0.357 |
| 355 | GATA9 | SLD5 | 3702.O82632 | 3702.Q6NQP5 | 0.431 |
| 356 | GATA9 | GUS3 | 3702.O82632 | 3702.Q9FZP1 | 0.574 |
| 357 | GBF1 | HDG11 | 3702.P42774 | 3702.Q9FX31 | 0.165 |
| 358 | GBF1 | PDF2 | 3702.P42774 | 3702.Q93V99 | 0.258 |
| 359 | GL2 | PIN3 | 3702.P46607 | 3702.Q9S7Z8 | 0.161 |
| 360 | GL2 | HAT4 | 3702.P46607 | 3702.Q05466 | 0.197 |
| 361 | GL2 | Q8S8Q2_ARATH | 3702.P46607 | 3702.Q8S8Q2 | 0.227 |
| 362 | GL2 | MPK3 | 3702.P46607 | 3702.Q39023 | 0.309 |
| 363 | GL2 | SPY | 3702.P46607 | 3702.Q96301 | 0.392 |
| 364 | GL2 | HDG11 | 3702.P46607 | 3702.Q9FX31 | 0.412 |
| 365 | GUS3 | PRA1B4 | 3702.Q9FZP1 | 3702.O80915 | 0.37 |
| 366 | GUS3 | SLD5 | 3702.Q9FZP1 | 3702.Q6NQP5 | 0.55 |
| 367 | GUS3 | STP14 | 3702.Q9FZP1 | 3702.Q8GW61 | 0.54 |
| 368 | HAT14 | HAT3 | 3702.P46665 | 3702.P46602 | 0.75 |
| 369 | HAT14 | HAT22 | 3702.P46665 | 3702.P46604 | 0.816 |
| 370 | HAT14 | HDG11 | 3702.P46665 | 3702.Q9FX31 | 0.228 |
| 371 | HAT14 | HAT4 | 3702.P46665 | 3702.Q05466 | 0.592 |
| 372 | HAT22 | HAT3 | 3702.P46604 | 3702.P46602 | 0.718 |
| 373 | HAT22 | PDF2 | 3702.P46604 | 3702.Q93V99 | 0.153 |
| 374 | HAT22 | SAG113 | 3702.P46604 | 3702.Q9FIF5 | 0.181 |
| 375 | HAT22 | NAC072 | 3702.P46604 | 3702.Q93VY3 | 0.277 |
| 376 | HAT22 | HDG11 | 3702.P46604 | 3702.Q9FX31 | 0.337 |
| 377 | HAT22 | HAT4 | 3702.P46604 | 3702.Q05466 | 0.655 |
| 378 | HAT3 | PIN3 | 3702.P46602 | 3702.Q9S7Z8 | 0.214 |
| 379 | HAT3 | HDG11 | 3702.P46602 | 3702.Q9FX31 | 0.25 |
| 380 | HAT3 | PDF2 | 3702.P46602 | 3702.Q93V99 | 0.317 |
| 381 | HAT3 | HAT4 | 3702.P46602 | 3702.Q05466 | 0.653 |
| 382 | HAT4 | PDF2 | 3702.Q05466 | 3702.Q93V99 | 0.361 |
| 383 | HAT4 | PIN3 | 3702.Q05466 | 3702.Q9S7Z8 | 0.404 |
| 384 | HAT4 | HDG11 | 3702.Q05466 | 3702.Q9FX31 | 0.632 |
| 385 | HDG11 | Q8S8Q2_ARATH | 3702.Q9FX31 | 3702.Q8S8Q2 | 0.411 |
| 386 | HDG11 | HDG2 | 3702.Q9FX31 | 3702.Q94C37 | 0.465 |
| 387 | HDG2 | MPK3 | 3702.Q94C37 | 3702.Q39023 | 0.259 |
| 388 | HDG2 | Q8S8Q2_ARATH | 3702.Q94C37 | 3702.Q8S8Q2 | 0.523 |
| 389 | HDG2 | PIN3 | 3702.Q94C37 | 3702.Q9S7Z8 | 0.378 |
| 390 | LTI65 | PRA1B4 | 3702.Q04980 | 3702.O80915 | 0.194 |
| 391 | LTI65 | MPK3 | 3702.Q04980 | 3702.Q39023 | 0.359 |
| 392 | LTI65 | SAG113 | 3702.Q04980 | 3702.Q9FIF5 | 0.75 |
| 393 | LTI65 | NAC072 | 3702.Q04980 | 3702.Q93VY3 | 0.855 |
| 394 | MPK3 | NAC072 | 3702.Q39023 | 3702.Q93VY3 | 0.168 |
| 395 | MPK3 | PDF2 | 3702.Q39023 | 3702.Q93V99 | 0.226 |
| 396 | MPK3 | SAG113 | 3702.Q39023 | 3702.Q9FIF5 | 0.703 |
| 397 | NAC072 | SAG113 | 3702.Q93VY3 | 3702.Q9FIF5 | 0.728 |
| 398 | NRAMP2 | NRAMP6 | 3702.Q9C6B2 | 3702.Q9S9N8 | 0.226 |
| 399 | NRAMP3 | VTL5 | 3702.Q9SNV9 | 3702.Q9LSF6 | 0.495 |
| 400 | NRAMP3 | NRAMP6 | 3702.Q9SNV9 | 3702.Q9S9N8 | 0.224 |
| 401 | PDF2 | Q8S8Q2_ARATH | 3702.Q93V99 | 3702.Q8S8Q2 | 0.516 |
| 402 | PDF2 | PIN3 | 3702.Q93V99 | 3702.Q9S7Z8 | 0.396 |
| 403 | PIN3 | Q8S8Q2_ARATH | 3702.Q9S7Z8 | 3702.Q8S8Q2 | 0.365 |
| 404 | PIN3 | WAG1 | 3702.Q9S7Z8 | 3702.Q9C8M5 | 0.794 |
| 405 | PRA1B4 | STP14 | 3702.O80915 | 3702.Q8GW61 | 0.198 |
| 406 | PRA1B4 | SLD5 | 3702.O80915 | 3702.Q6NQP5 | 0.261 |
| 407 | Q8S8Q2_ARATH | Q9LSC7_ARATH | 3702.Q8S8Q2 | 3702.Q9LSC7 | 0.239 |
| 408 | Q9LSC7_ARATH | SPY | 3702.Q9LSC7 | 3702.Q96301 | 0.237 |
| 409 | Q9LSC7_ARATH | T14D3.150 | 3702.Q9LSC7 | 3702.Q9M1T8 | 0.284 |
| 410 | SLD5 | STP14 | 3702.Q6NQP5 | 3702.Q8GW61 | 0.344 |
| 411 | SPY | T14D3.150 | 3702.Q96301 | 3702.Q9M1T8 | 0.191 |
| 412 | T14D3.150 | WAG1 | 3702.Q9M1T8 | 3702.Q9C8M5 | 0.576 |

**Table S5** Annotation of the interacting proteins of SaHDZ22 in yeast screening.

| NO | Gene length | CDS and amino acid length | Annotation |
| --- | --- | --- | --- |
| A1 | 1168 bp | 828 bp；275 aa | hypothetical protein FOXB_00843 |
| A2 | 1210 bp | 951 bp；316 aa | bifunctional 3,4-dihydroxy-2-butanone-4-phosphate synthase/GTP cyclohydrolase II |
| A5 | 832 bp | 543 bp；180 aa | hypothetical protein Poli38472_009718 |
| A12 | 823 bp | 774 bp；257 aa | Y-family of DNA polymerases |
| A13 | 1113 bp | 798 bp；265 aa | protein SRC2-like isoform X1 |
| A16 | 1114 bp | 540 bp；179 aa | Adenylate kinase isoenzyme homolog |
| A18 | 754 bp | 705 bp；234 aa | Tetratricopeptide repeat (TPR)-like superfamily protein |

**Figure S1.** Prediction of SaHDZ interacting protein networks in *S. alopecuroides*.

**Figure S2** Screening of SaHDZ22 interacting proteins. (A) Screening of yeast monoclones; (B) PCR detection and screening of protein genes.
